# Comprehensive Understanding of Elemental Doping and Substitution of Ni‐Rich Cathode Materials for Lithium‐Ion Batteries via In Situ Operando Analyses

**DOI:** 10.1002/smsc.202400165

**Published:** 2024-07-08

**Authors:** Yun Seong Byeon, Wontae Lee, Sangbin Park, Dongil Kim, Jaewoo Jung, Min‐Sik Park, Won‐Sub Yoon

**Affiliations:** ^1^ Department of Advanced Materials Engineering for Information and Electronics Integrated Education Institute for Frontier Science & Technology (BK21 Four) Kyung Hee University 1732 Deogyeong‐daero Giheung‐gu Yongin 17104 Republic of Korea; ^2^ Department of Chemistry Education Kyungpook National University 80, Daehak‐ro Buk‐gu Daegu 41566 Republic of Korea; ^3^ Department of Energy Science Sungkyunkwan University Suwon 16419 Republic of Korea; ^4^ SKKU Institute of Energy Science and Technology (SIEST) Sungkyunkwan University Suwon 16419 Republic of Korea

**Keywords:** doping/substitution, in situ operando analysis, lithium‐ion batteries, reaction mechanisms

## Abstract

This review explores the challenges and advancements in the development of high‐energy lithium‐ion batteries (LIBs), particularly focusing on the electrochemical and structural stability of Ni‐rich cathode materials. Despite their potential to increase the energy density of LIBs, these cathode materials encounter issues such as irreversible phase transitions and structural degradation during cycling, which ultimately affect their electrochemical performance. Elemental doping/substitution has emerged as promising strategies to address these challenges. However, the precise mechanisms underlying their performance enhancement remain unclear. The objective is to elucidate the complex reaction mechanisms triggered by doping and substitution in Ni‐rich cathode materials by employing in situ operando analyses to uncover their effects on electrochemical behavior and structural integrity during cycling. This comprehensive investigation aims to clarify the roles of elemental dopants or substituents in the crystal structures of Ni‐rich cathode materials, thereby offering valuable insights for the structural engineering of cathode materials in high‐energy LIBs. By elucidating these intricate mechanisms, this review provides a practical roadmap for future research and significantly contributes to LIB technology by guiding material design and optimization strategies in the development of advanced LIBs.

## Graphical abstract

This review emphasizes employing in situ operando techniques to understand the impact of doping/substitution on nickel‐rich cathode materials, showcasing their efficacy in revealing performance enhancement mechanisms. By integrating these methods, it provides a valuable resource for designing optimized energy storage materials, contributing significantly to material science field by illustrating the combined benefits of doping/substitution strategies and in situ operando analyses.

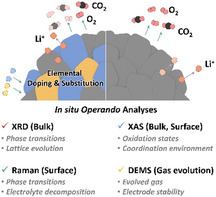

## 1 Introduction

The development of high‐energy lithium‐ion batteries (LIBs) is critical for advancing energy storage technologies, and nickel (Ni)‐rich cathode materials have emerged as promising candidates due to their potential to significantly increase energy density.^[^ ^1^, ^2^, ^3^ ^]^ However, their practical application is often limited by challenges such as irreversible phase transitions and structural degradation during cycling, resulting in diminished electrochemical performance.^[^ ^4^, ^5^ ^]^ Recently, various strategies, including elemental doping/substitution, have been employed to address these issues and enhance the stability and performance of Ni‐rich cathode materials.

There is an extensive research on both the elemental doping/substitution of Ni‐rich cathode materials and the utilization of in situ characterization methods.^[^ ^6^, ^7^, ^8^ ^]^ In particular, elemental doping/substitution strategies have been extensively explored to improve structural integrity and electrochemical performance, while in situ and operando techniques have provided valuable real‐time insights into the dynamic processes occurring within Ni‐rich cathode materials during operation. Despite considerable efforts, there remains a need for a comprehensive understanding of how doping/substitution directly influences electrochemical performance of the Ni‐rich cathode materials, as revealed through in situ operando analyses.

The primary focus of this review is to bridge these two areas by demonstrating how in situ operando techniques can be effectively used to understand the mechanisms by which doping/substitution enhances the electrochemical performance of Ni‐rich cathode materials. By using Ni‐rich layered cathodes as an example, we illustrate how changes in physical and chemical properties induced by doping/substitution can be systematically investigated using advanced in situ technologies. These techniques allow for real‐time monitoring of structural, electronic, and electrochemical changes, providing critical insights into the effects of doping/substitution on material properties.

Our review emphasizes the application of in situ operando methodologies specifically to Ni‐rich cathode materials, showcasing their power in elucidating the phenomena underlying performance improvements. By offering a well‐organized integration of these approaches, we aim to provide a valuable resource for researchers seeking to optimize various cathode materials as well as cell design of high‐energy LIBs. Ultimately, we believe our review contributes significantly to the field by highlighting the synergistic benefits of combining doping/substitution strategies with cutting‐edge in situ operando analyses.

### 1.1 Development of Ni‐Rich Cathode Materials for High‐Energy LIBs

LIBs have contributed significantly to the advancement of electric vehicles (EVs) owing to their high energy density and impressive cycle life. These characteristics have enabled the successful implementation of LIBs in EVs.^[^ ^9^, ^10^, ^11^, ^12^, ^13^ ^]^ However, for EVs to gain broader market acceptance, the current LIB technology faces significant challenges, including issues related to production costs, safety concerns, and limited driving range. Therefore, there is an urgent need to enhance the energy density, cycling durability, and safety of LIBs.^[^ ^14^, ^15^, ^16^, ^17^, ^18^ ^]^ Graphite, a commercial anode material, sets a high‐performance benchmark with a discharge capacity of 360 mAh g^−1^. In contrast, cathode materials typically exhibit lower capacities, reduced cycle life, suboptimal thermal properties, and higher production costs. Consequently, current research on LIBs has focused on tailoring commercial cathode materials or discovering new materials to enhance the overall performance of LIBs.^[^ ^3^, ^19^, ^20^, ^21^, ^22^ ^]^ Among various materials, layered oxides based on LiNiO_2_ have attracted significant attention due to their high capacity and favorable electrochemical properties. It was proposed as electrode materials for LIBs in 1985.^[^ ^23^ ^]^ However, the pure LiNiO_2_ phase suffers from structural instability and rapid capacity fading during cycling, necessitating modifications to enhance its performance.^[^ ^24^, ^25^, ^26^ ^]^ A promising approach to improve the electrochemical properties of LiNiO_2_ involves partial substitution of Ni with other transition metals or elements. This strategy has led to the investigation of various solid solutions within the LiNi_1−*x*_M*_x_* O_2_ (M = Co, Mn, Al) systems. For instance, a study by C. Delmas et al. explored the crystal chemistry and electrochemical behavior of the LiNi_1−*x*_Co*_x_* O_2_ system, demonstrating improvements in structural stability and cycling performance with Co substitution.^[^ ^27^ ^]^ Similarly, Rossen et al. examined the effects of Mn substitution in LiNi_1−*x*_Mn*_x_* O_2_, revealing enhancements in both the crystal structure and electrochemical properties.^[^ ^28^ ^]^ The inclusion of Mn was found to contribute to better capacity retention and thermal stability. Another important study by Zhong and von Sacken, along with Ohzuku, focused on the LiNi_1−*x*_Al*_x_* O_2_ solid solution, highlighting the synthesis, structural characterization, and electrochemical performance of these materials.^[^ ^29^, ^30^ ^]^ The introduction of Al was shown to mitigate the issues of structural degradation and capacity loss. Building upon these binary systems, Liu et al. investigated a ternary system, LiNi_1−*x*−y_Co*_x_* Mn_y_O_2_, which combines the benefits of both Co and Mn substitution.^[^ ^31^ ^]^ This approach aimed to optimize the balance between capacity, stability, and safety. Further advancements were made by K. Matsumoto et al. who synthesized and characterized the electrochemical properties of LiNi_1−*x*−y_Co*_x_* Al_y_O_2_, incorporating Al alongside Co to further enhance the material's performance and stability.^[^ ^32^ ^]^ These studies collectively contribute to the understanding and development of advanced LiNiO_2_‐based electrode materials, paving the way for the design of next‐generation LIBs with superior capacity, stability, and safety features. In particular, the layered Li[Ni_*x*_Co_*y*_Mn(Al)_1–*x*−*y*_]O_2_ (known as NCM or NCA) stand out as the most promising candidate for high‐energy LIBs due to their substantial theoretical capacity (≈280 mAh g^−1^) and relatively high average operating potential of 3.6 V versus Li/Li^+^. These attributes make them frontrunners in addressing the limitations of current LIBs for EV applications.^[^ ^33^, ^34^, ^35^, ^36^, ^37^, ^38^, ^39^ ^]^

**Figure 1:**
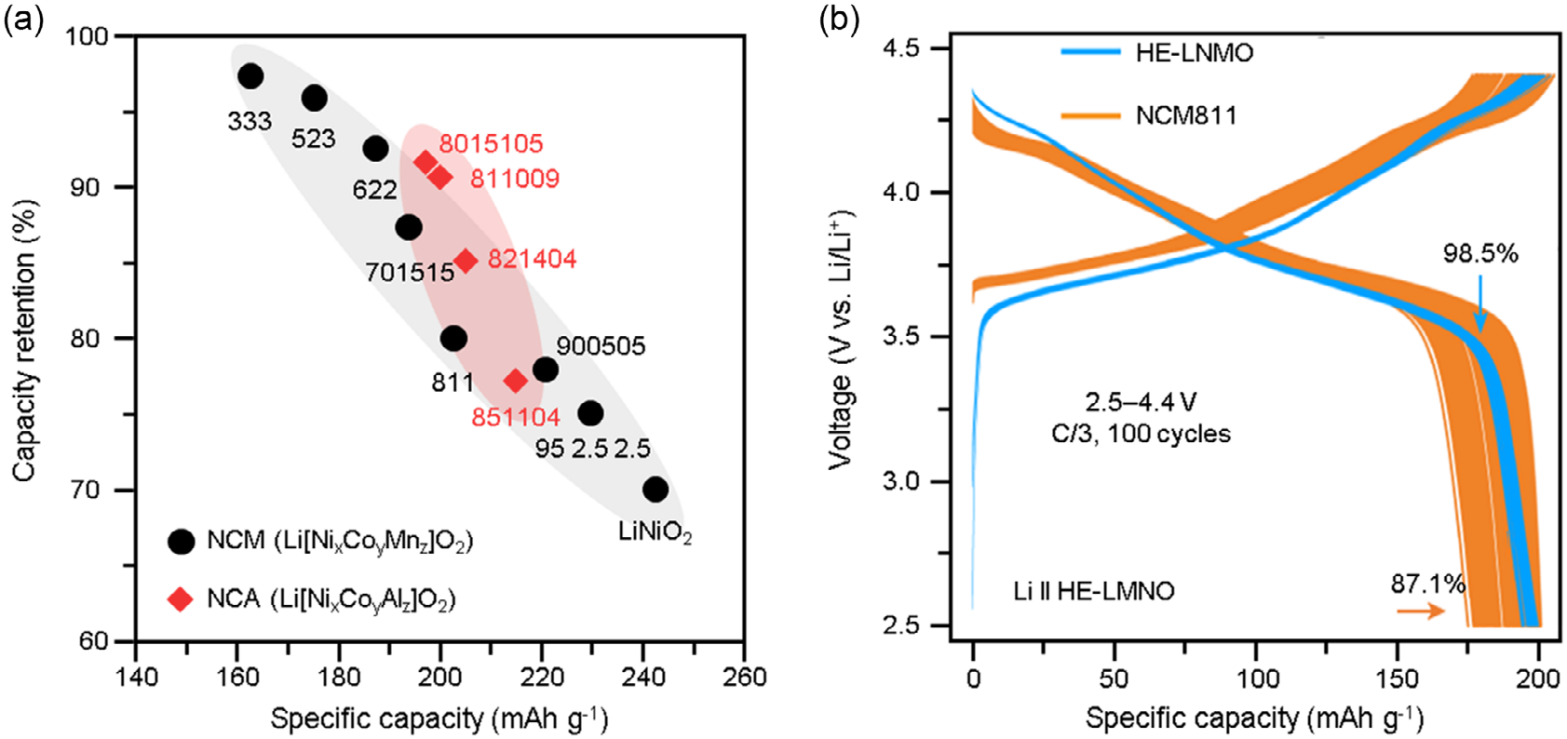
a) Specific capacity versus cycling stability plot of various layered cathodes. Reproduced with permission.^[^ ^50^ ^]^ Copyright 2017, American Chemical Society. b) Long‐cycle charge–discharge profiles of the half cells containing HE‐LNMO and NCM811 within 2.5–4.4 V versus Li/Li^+^ at the current density of *C*/3. Reproduced with permission.^[^ ^69^ ^]^ Copyright 2022, Springer Nature.

Liu et al. first reported the LiNi_0.5_Co_0.2_Mn_0.3_O_2_, LiNi_0.6_Co_0.2_Mn_0.2_O_2_, and LiNi_0.7_Co_0.2_Mn_0.1_O_2_ cathode materials for LIBs in 1990.^[^ ^31^ ^]^ Subsequently, various research groups, including Yoshio et al. and Ohzuku et al. developed LiNi_*x*_Co_1–*x*–*y*_Mn_*y*_O_2_ (0.1 ≤ *x* ≤ 0.5), demonstrating their excellent thermal and structural stability.^[^ ^40^, ^41^ ^]^ In 2006, Kim et al. successfully synthesized spherical Ni‐rich LiNi_0.8_Co_0.1_Mn_0.1_O_2_ and LiNi_0.8_Co_0.2_O_2_ cathode materials, reporting reversible capacities of ≈200 mAh g^−1^.^[^ ^42^ ^]^ Since then, numerous researchers have explored various compositions of Ni‐rich cathode materials, leading to increased reversible capacities.^[^ ^35^, ^43^, ^44^ ^]^ In other words, increasing the Ni concentration in Ni‐rich NCM and NCA cathode materials is a strategy to boosting their reversible capacity. However, this improvement comes with a tradeoff: a decrease in the structural and thermal stabilities of these cathode materials. This issue arises mainly from the surface degradation of the cathode particles, especially those with high Ni concentrations, such as NCM and NCA.^[^ ^4^, ^5^, ^45^, ^46^, ^47^ ^]^ The primary concern is the transformation of Ni^4+^ into a more stable yet electrically insulating Ni—O phase. This transformation occurs alongside the breakdown of the electrolyte on the cathode surface and the formation of microcracks owing to repeated charging and discharging. Microcracks resulting from inconsistent expansion and contraction within cathode particles highlight their inherent weakness. As microcracks propagate, they allow the electrolyte to penetrate deeply into the cathode particles, further damaging them by forming an insulating layer similar to that of Ni—O on the surface of the primary particles. This issue is particularly pronounced in cathode materials with high Ni concentrations.^[^ ^48^, ^49^, ^50^, ^51^, ^52^, ^53^, ^54^ ^]^ **Figure** **1**a highlights the relationship between specific capacity and capacity retention of NCM and NCA with varying Ni concentrations.^[^ ^50^ ^]^ Specific capacities were measured within a voltage range of 2.7–4.3 V versus Li/Li^+^, and capacity retentions were determined after 100 cycles. As previously mentioned, an increase in Ni concentration correlates with an enhanced capacity; for instance, LiNi_1/3_Co_1/3_Mn_1/3_O_2_ (NCM333) achieves a capacity of ≈160 mAh g^−1^, LiNi_0.6_Co_0.2_Mn_0.2_O_2_ (NCM622) ≈190 mAh g^−1^, LiNi_0.8_Co_0.1_Mn_0.1_O_2_ (NCM811) around 200 mAh g^−1^, and LiNi_0.85_Co_0.11_Al_0.04_O_2_ (NCA851104) about 215 mAh g^−1^. However, it is obvious that an increase in Ni concentrations also leads to performance during cycling, with capacity retentions of ≈98% for NCM333, 91% for NCM622, 80% for NCM811, and 78% for NCA851104. Despite these challenges, the demand for longer driving ranges in EVs continues to drive the development of Ni‐rich NCM cathodes, emphasizing the ongoing pursuit of a balance between reversible capacity and durability.^[^ ^55^, ^56^, ^57^ ^]^

### 1.2 Research on Improvement of Structural Stability of Ni‐Rich Cathode Materials: Elemental Doping and Substitution

Incorporating single or multiple elements into the crystal structures of Ni‐rich cathode materials has emerged as an effective strategy for mitigating undesirable capacity loss during cycling. This approach not only preserves the structural integrity of these cathode materials but also enhances lithium‐ion (Li^+^) transport within the structure.^[^ ^58^, ^59^, ^60^, ^61^, ^62^ ^]^ Elemental doping and substitution with various cations or anions have been successfully employed to resolve issues such as capacity fading, irreversible phase transitions, and microcrack formation, thereby enhancing the structural and thermal resilience as well as cycling performance.^[^ ^63^, ^64^, ^65^, ^66^, ^67^ ^]^

To enhance the structural integrity and prolong the cycling performance of Ni‐rich cathode materials, researchers have adopted elemental doping and substitution strategies. In practice, various elements, such as Na, Mg, Al, Ti, Zn, and F, each with distinct properties, have been explored to tailor the characteristics of Ni‐rich cathode materials.^[^ ^68^, ^69^, ^70^, ^71^, ^72^, ^73^, ^74^, ^75^, ^76^ ^]^ The precise integration of these elements into the crystal structures of host materials has been scientifically validated to reinforce the structural integrity and durability of the materials. Remarkably, even minor doping or substitution levels, often below 2 mol%, can substantially enhance the structural stability and cycling performance of cathode materials. These findings are detailed in Table S1, Supporting Information, which provides an extensive summary of the experimental outcomes and highlights the beneficial effects of strategic doping and substitutions on material characteristics. In practice, Zhang et al. reported the positive effects of high‐entropy doping with Ti, Mg, Nb, and Mo via the coprecipitation method.^[^ ^69^ ^]^ These multiple dopants effectively mitigated oxygen evolution due to pinning effects, reduced lattice expansion–contraction, and suppressed cation mixing and phase transitions to spinel/rock‐salt phases during cycling. As a result, LiNi_0.8_Mn_0.13_Ti_0.02_Mg_0.02_Nb_0.01_O_2_ (HE‐LNMO) demonstrated superior thermal and structural stability compared to commercial Ni‐rich cathodes. Consequently, the HE‐LNMO cathode exhibited a stable cycle performance, retaining 98.5% of initial capacity after 100 cycles within 2.5–4.4 V versus Li/Li^+^ at a current density of *C*/3 (Figure 1b). According to Zheng et al. LiNi_0.9_Mn_0.1_O_2_ (NM) cathodes are classified as Ni‐rich cathode materials. In this case, the occurrence of Li/Ni superexchange interaction becomes more prevalent, facilitating the transition of Ni^2+^ into the Li layer during cycling. Consequently, this leads to severe cation disorder with a decrease in Li^+^ diffusion coefficients. However, substituting elements like Co/Al, which do not have unpaired spins, into the Ni‐rich NM cathode can significantly increase the formation energy of Li/Ni exchange, thereby suppressing cation disorder.^[^ ^77^ ^]^ In practice, Xiao et al. conducted an in‐depth investigation of the mechanisms underlying the enhanced kinetics observed with Co/Al substitution in NM cathode materials through meticulous coprecipitation synthesis (Figure S1a, Supporting Information).^[^ ^78^ ^]^ The LiNi_0.84_Mn_0.10_Co_0.03_Al_0.03_O_2_ (CA‐NM) cathode material exhibited exceptional enhancements, particularly in rate capability and long‐term cycling stability. In practice, the CA‐NM cathode demonstrated a remarkable capacity retention rate of 76.8% under rapid charging conditions at a 5 C rate over 200 cycles. This outstanding electrochemical performance can be attributed to a significant reduction in the degree of cation disorder within the structure. A thorough analysis revealed that Co/Al substitution led to pronounced suppression of cation disorder, resulting in a substantial decrease in the occurrence of irreversible phase transitions, particularly the transition of Ni^2+^ into Li layers during cycling. Ryu et al. reported the positive effects of Mo doping into LiNi_0.94_Co_0.03_Mn_0.02_Al_0.01_O_2_ (NCMA94) to establish an optimal cathode structure. The Mo refined the primary particle size of the NCMA94 cathode material by enhancing the grain texture stability across various lithiation temperatures and improving the crystallinity.^[^ ^79^ ^]^ Consequently, this adjustment significantly enhanced cycling stability and mechanical resistance to microcracking. The LiNi_0.935_Co_0.025_Mn_0.02_Al_0.01_Mo_0.01_O_2_ (Mo1‐NCMA94) cathode, treated at 750 °C, exhibited superior performance, retaining 77.9% capacity (@ 1000 cycles), which is an improvement over the 64.7% retention of a pristine NCMA94 cathode.

Anion doping and substitution represent another effective strategy for enhancing the electrochemical performance of Ni‐rich cathode materials and addressing challenges such as cation mixing and oxygen evolution.^[^ ^80^, ^81^, ^82^, ^83^ ^]^ For example, Kim et al. successfully synthesized an F‐doped NCM811 cathode material through a solid‐state reaction.^[^ ^84^ ^]^ This process involved substituting F for O in the given structure, strengthening the bonds between transition metals and F. Consequently, this modification increased the *c*‐lattice parameter from 14.16 to 14.18 Å, leading to a notable enhancement in Li^+^ diffusivity. Specifically, the Li^+^ diffusion coefficient increased from 1.24 × 10^−10^ to 1.35 × 10^−10^ cm^2^ s^−1^ in the F‐doped cathode (NCMF). As a result, the NCMF cathode demonstrated a superior discharge capacity of 201 mAh g^−1^ and maintained 92.0% of its initial capacity over 100 cycles, whereas the NCM cathode showed a discharge capacity of 179 mAh g^−1^ and 81.0% capacity retention after 100 cycles (Figure S1b, Supporting Information).

## 2 In Situ Operando Analytical Methodology

**Figure 2:**
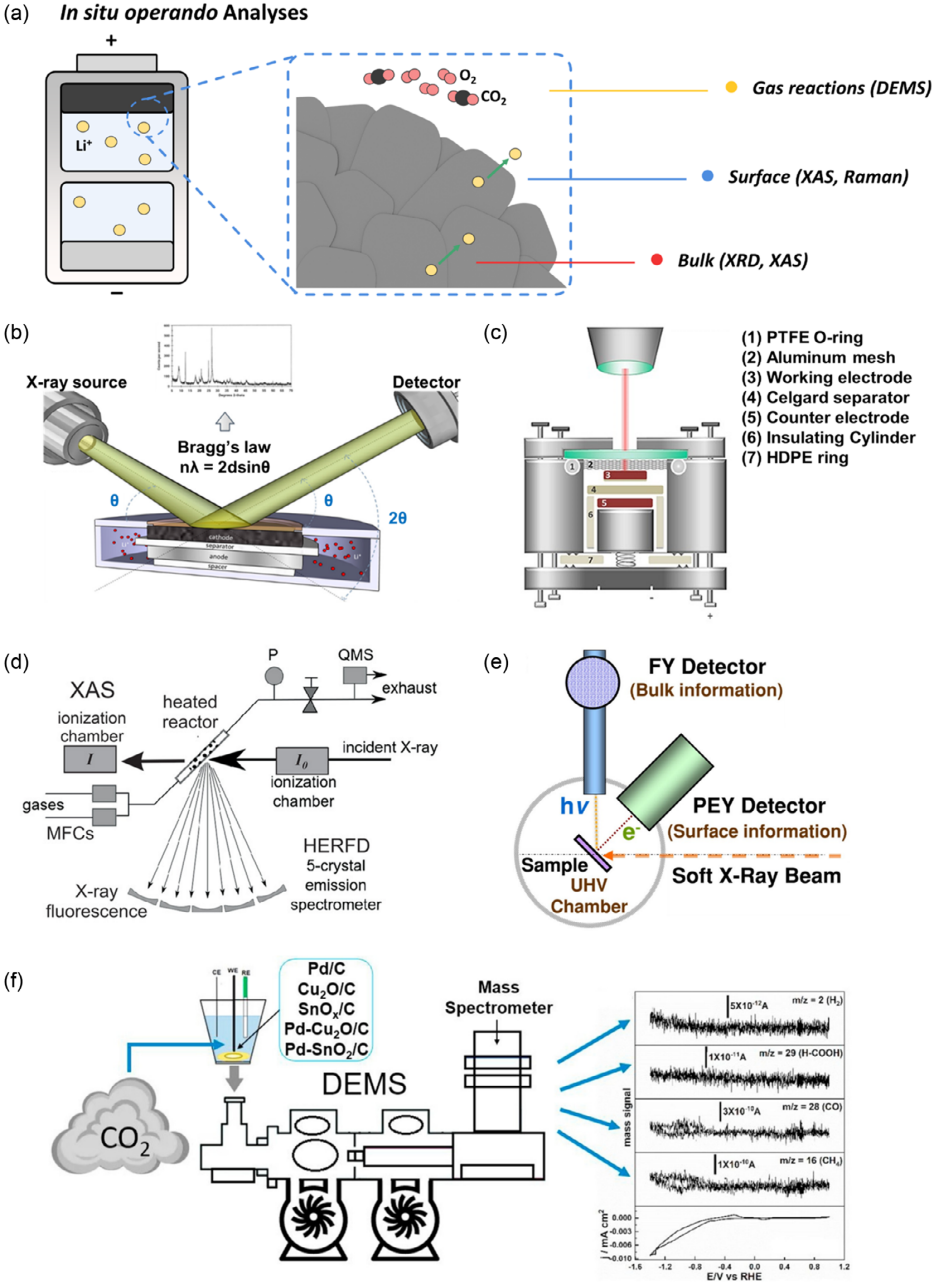
a) Summary of the in situ operando techniques. Mechanisms of operando b) XRD, Reproduced with permission.^[^ ^113^ ^]^ Copyright 2020, MDPI; c) Raman, Reproduced with permission.^[^ ^118^ ^]^ Copyright 2018, Frontiers; d) hard XAS, Reproduced with permission.^[^ ^138^ ^]^ Copyright 2021, Wiley; e) soft XAS, Reproduced with permission.^[^ ^156^ ^]^ Copyright 2014, Springer Nature; and f) DEMS, Reproduced with permission.^[^ ^147^ ^]^ Copyright 2021, Elsevier.

Ongoing efforts to enhance the electrochemical performance of LIBs, focusing on energy density, cycling performance, and durability, have made significant progress through the strategic doping or substitution of Ni‐rich cathode materials.^[^ ^58^, ^59^, ^60^, ^68^, ^69^ ^]^ These advancements underscore a broader imperative within LIB technology, necessitating not only the optimization of existing cathode materials but also the integration of novel materials with superior electrochemical properties.^[^ ^85^, ^86^, ^87^, ^88^ ^]^ Such endeavors demand a nuanced understanding of the intricate relationship between structural alterations in cathode materials and their consequential effects on the electrochemical performance during operation.^[^ ^89^, ^90^, ^91^, ^92^, ^93^ ^]^ Moreover, it is crucial to understand the degradation mechanisms during cycling and aging, as well as thermal decomposition under heat exposure.^[^ ^94^, ^95^ ^]^ For a comprehensive understanding, analyses must span multiple scales and employ a diverse array of characterization techniques, both individually and synergistically. Recent developments have led to significant improvements in characterization methods for LIBs, which are suitable for both ex situ and in situ and operando conditions. As a characterization method for the Ni‐rich cathode materials, ex situ analyzing techniques offer merits in high resolution and precision. They provide critical insights into structural evolution, redox mechanisms, solid–electrolyte interphase formation, side reactions, and Li^+^ transport behaviors under various operational conditions.^[^ ^96^, ^97^, ^98^, ^99^, ^100^ ^]^ However, the retrospective nature of ex situ characterization methods limits their capacity to explore the kinetic characteristics of materials, such as intricate structural alterations and transitional states during charge–discharge cycles or thermal events. Furthermore, the vulnerability of working electrodes to air and moisture may affect the accuracy of ex situ findings. This affects the understanding of electrochemical reactions, such as valence changes and surface or interfacial reactions, which may not accurately reflect the structural and electronic information of the target materials.^[^ ^101^, ^102^, ^103^ ^]^ Consequently, gathering data under realistic operational conditions is imperative for the strategic development of LIBs. This highlights the critical role of in situ operando characterization techniques in providing a genuine understanding of electrochemical processes and material dynamics under operational stresses.^[^ ^104^ ^]^ Among the diverse in situ and operando analytical methodologies available, this section describes four techniques that are effective for investigating phenomena manifesting within the bulk and on the surface of cathode materials, as well as those related to gas evolution (**Figure** **2**a and **Table** **1**).

**Table 1:** In situ operando characterization techniques applied to Ni‐rich cathode materials.

Technique	Experimental subject	Major discoveries
XRD	Al‐doped LiNi_0.80_Co_0.15_Mn_0.05_O_2_^[^ ^54^ ^]^	Enhanced thermal stability at the charged state via Al doping
	Ti‐doped LiNi_0.8_Co_0.1_Mn_0.1_O_2_^[^ ^162^ ^]^	Alleviated lattice change during Li^+^ removal due to the stabilizing effect of Ti
	LiNi_0.8_Mn_*x*_Al_*y*_O_2_^[^ ^163^ ^]^	Improved structural reversibility of the Co‐free cathode due to the influence of Al and cation vacancies
	Mo and F codoped LiNi_0.92_Co_0.04_Mn_0.04_O_2_^[^ ^164^ ^]^	Mitigated lattice shrinkage along the *c*‐axis as a result of a surficial antisite defect layer
	Mg and Al codoped LiNi_0.95_Co_0.03_Al_0.01_Mg_0.01_O_2_^[^ ^61^ ^]^	Slow lattice contraction during charging of concentration‐gradient Mg–Al doping
Raman spectroscopy	LiNi_0.85−*x*_Co_0.15_Al_*x*_O_2_^[^ ^165^ ^]^	Enhanced stability at the interface of the cathode material and sulfide solid electrolyte through greater Al substitution for Ni
	B‐doped LiNi_0.8_Co_0.1_Mn_0.1_O_2_^[^ ^166^ ^]^	Curbed oxygen release during high‐voltage cycling via the piezoelectric impact of Li_2_B_4_O_7_
	Mg–Ti codoped/surface‐treated LiNi_0.8_Co_0.1_Mn_0.1_O_2_^[^ ^167^ ^]^	Reversible oxygen redox reaction during high‐voltage cycling through the inhibited outward movement of oxidized oxygen anions
	LiNi_0.84_Co_0.10_Mn_0.06_O_2_^[^ ^168^ ^]^	Thermal decomposition of the surface structure under fully charged conditions
	LiNi_0.92_Co_0.04_Mn_0.04_O_2_^[^ ^169^ ^]^	Abrupt lattice contraction during the H2–H3 phase transition in the charging process
XAS	LiNi_0.88_Co_0.1_Al_0.02_O_2_^[^ ^157^ ^]^	Anionic redox reaction at deep delithiation state investigated by the change in valence state and atomic distances
	LiNi_0.8_Co_0.1_Mn_0.1_O_2_^[^ ^170^ ^]^	Lattice O oxidation explored through the elongation of the Ni—O bond
	LiNiO_2_^[^ ^171^ ^]^	Alteration in the oxidation state and local structure during the cathode material synthesis from its precursor
	LiNi_0.6_Co_0.2_Mn_0.2_O_2_^[^ ^172^ ^]^	Inhibition of oxygen release at elevated temperatures via oligomer addition
	Li_0.33_Ni_0.8_Co_0.15_Al_0.05_O_2_^[^ ^156^ ^]^	NiO‐like rock‐salt phase formation at high temperatures in the charged cathode material
DEMS	LiNi_0.85_Co_0.10_Mn_0.05_O_2_^[^ ^173^ ^]^	Restricted CO_2_ evolution during cycling by hybrid ALD coating
	LiNi_0.85_Mn_0.07_Co_0.05_Al_0.03_O_2_	Need for further delithiation after H2–H3 phase transition and breach formation for gas release
	LiNi_0.92_Mn_0.02_Co_0.04_Al_0.02_O_2_^[^ ^174^ ^]^	
	Zr‐doped LiNi_0.92_Co_0.04_Mn_0.04_O_2_^[^ ^158^ ^]^	Reduction in oxygen gas release and electrolyte decomposition achieved by Zr doping
	Ta‐doped LiNi_0.88_Co_0.10_Al_0.02_O_2_^[^ ^57^ ^]^	Suppression of oxygen loss during high‐voltage cycling via Ta doping

### 2.1 In Situ Operando X‐Ray Diffraction Techniques

X‐ray diffraction (XRD) is extensively used to obtain crystallographic information on materials, operating on the principle of X‐ray scattering to detect distinctive diffraction patterns at specific angles, known as Bragg angles.^[^ ^105^, ^106^, ^107^, ^108^, ^109^ ^]^ Through the precise analysis of peak positions and intensities, a wealth of structural information can be extracted, such as the exact crystal structure and alterations in the atomic positions after elemental doping. In practical in situ operando experiments, most cells adopt the modified coin‐cell designs featuring drilled holes sealed with X‐ray transparent windows.^[^ ^110^, ^111^, ^112^ ^]^ Synchrotron X‐ray sources, known for their high intensity and significant photon energy, are particularly advantageous for in‐depth in situ operando investigations due to their deeper penetration, shorter measurement durations, and enhanced signal resolution.^[^ ^113^, ^114^ ^]^ With the deep penetration depth and sensitivity, in situ operando XRD enables the exploration of structural alterations, phase transition, and reaction dynamics under various electrochemical conditions. Thus, XRD plays an essential role in advancing battery technology by optimizing material properties and electrochemical processes (Figure 2b).

### 2.2 In Situ Operando Raman Techniques

Raman spectroscopy, developed after the discovery of Raman scattering in 1928, is a valuable tool for analyzing the structural and chemical properties of materials based on their molecular vibrations.^[^ ^115^ ^]^ When a laser interacts with a target material, scattered light produces a Raman spectrum reflecting the unique atomic vibrational modes of the sample.^[^ ^116^, ^117^, ^118^ ^]^ Raman spectroscopy is particularly useful for investigating amorphous materials with poor crystallinity, and its noninvasive nature allows for repeated examination. In the in situ operando analysis of Ni‐rich cathode materials, this technique enables real‐time examination of reaction kinetics,^[^ ^119^ ^]^ phase transitions,^[^ ^120^ ^]^ compositional changes,^[^ ^121^, ^122^ ^]^ and interfacial phenomena^[^ ^123^ ^]^ without interrupting cell operation. Tailored cell designs, such as coin‐cell^[^ ^124^, ^125^, ^126^ ^]^ and pouch‐cell configurations^[^ ^120^, ^127^, ^128^ ^]^ equipped with transparent windows for laser access, are necessary (Figure 2c). Raman spectroscopy offers several inherent benefits for the operando analysis of Ni‐rich cathode materials, including: 1) minimally invasive light probing; 2) submicrometer resolution for examining individual cathode particles using microscopic techniques; 3) compatibility with common window materials transparent to visible light; 4) rapid spectrum acquisition times for timely data collection; and 5) moderation of Raman signals from strong infrared‐absorbing substances like electrolyte solvents.^[^ ^129^, ^130^, ^131^ ^]^

### 2.3 In Situ Operando X‐Ray Absorption Spectroscopy

X‐ray absorption spectroscopy (XAS) is an indispensable technique for investigating Ni‐rich cathode materials, providing insights into the electronic characteristics and local arrangement of elements, such as valence state, coordination symmetry, bond lengths, and the degree of disorder.^[^ ^132^, ^133^, ^134^, ^135^ ^]^ This technique involves X‐ray absorption, where a core electron of specific atoms is promoted to a higher energy state.^[^ ^136^ ^]^ XAS is typically categorized into two types based on the energy of X‐ray: hard XAS (over 5 keV) and soft XAS (below 1 keV), as illustrated in Figure 2d,e, respectively.

Hard XAS (Figure 2d) is suitable for studying the bulk properties, and the setup for in situ operando hard XAS experiments can mirror that of in situ operando XRD, with modifications to allow X‐ray penetration.^[^ ^137^, ^138^, ^139^ ^]^ Thus, it is commonly used to investigate atoms with high atomic numbers by exciting the 1*s* electron to vacant *p* orbitals involving X‐ray absorption near‐edge structure (XANES) and extended X‐ray absorption fine structure (EXAFS).^[^ ^140^ ^]^ XANES identifies oxidation states and local symmetry, while EXAFS provides detailed information about the local chemical environment, bonding state, and structural disorder.^[^ ^141^, ^142^ ^]^

On the other hand, soft XAS captures the electron transitions, particularly in transition metal L‐/M‐edge and light element K‐edge spectra (Figure 2e). The relatively low energy leads to reduced penetration depths, making it surface‐sensitive up to hundreds of nanometers. Therefore, this method is advantageous for investigating chemical bonding, molecular geometry, and electronic configurations on the surface of Ni‐rich cathode materials.^[^ ^143^, ^144^, ^145^ ^]^ However, designing electrochemical cells for in situ operando analysis is challenging due to the shallow penetration depth, requiring bespoke setups to expose electrodes to X‐rays while avoiding interference from cell components.^[^ ^146^ ^]^

### 2.4 In Situ Operando Differential Electrochemical Mass Spectrometry Techniques

In situ operando characterization of evolved gases is also crucial for understanding reaction mechanisms and ensuring the safety of LIBs. Differential electrochemical mass spectrometry (DEMS) is widely used for this purpose.^[^ ^147^, ^148^ ^]^ Following the pioneering works of Bruckenstein et al.^[^ ^149^ ^]^ and Wolter et al.^[^ ^150^ ^]^ DEMS has become a key technique for assessing the composition and generation rates of gaseous species in electrochemical systems. It combines gas chromatography (GC) and mass spectrometry (MS), where evolved gas species arrive at the detector at varying times based on their mass‐to‐charge ratio (*m*/*z*). Coupling mass spectral data with GC‐derived retention times helps determine the relative concentrations or partial pressures of targeted gas species. In DEMS experiments, the design of electrochemical cell is crucial for facilitating the effective flow of gases.^[^ ^151^ ^]^ A carrier gas transports evolved gases from the electrochemical process to the mass spectrometer. A standard DEMS setup includes a test cell, a porous PTFE membrane for gas diffusion, and a vacuum line connecting to the mass spectrometer. The integration of the electrochemical system with the mass spectrometer enables DEMS to serve as an effective tool for continuous gas monitoring throughout the charge and discharge processes of LIBs (Figure 2f).^[^ ^152^ ^]^

## 3 Elucidation of Doping and Substitution Mechanisms of Ni‐Rich Cathode Materials Based on In Situ Operando Analytical Techniques

### 3.1 In Situ Operando XRD Techniques

**Figure 3:**
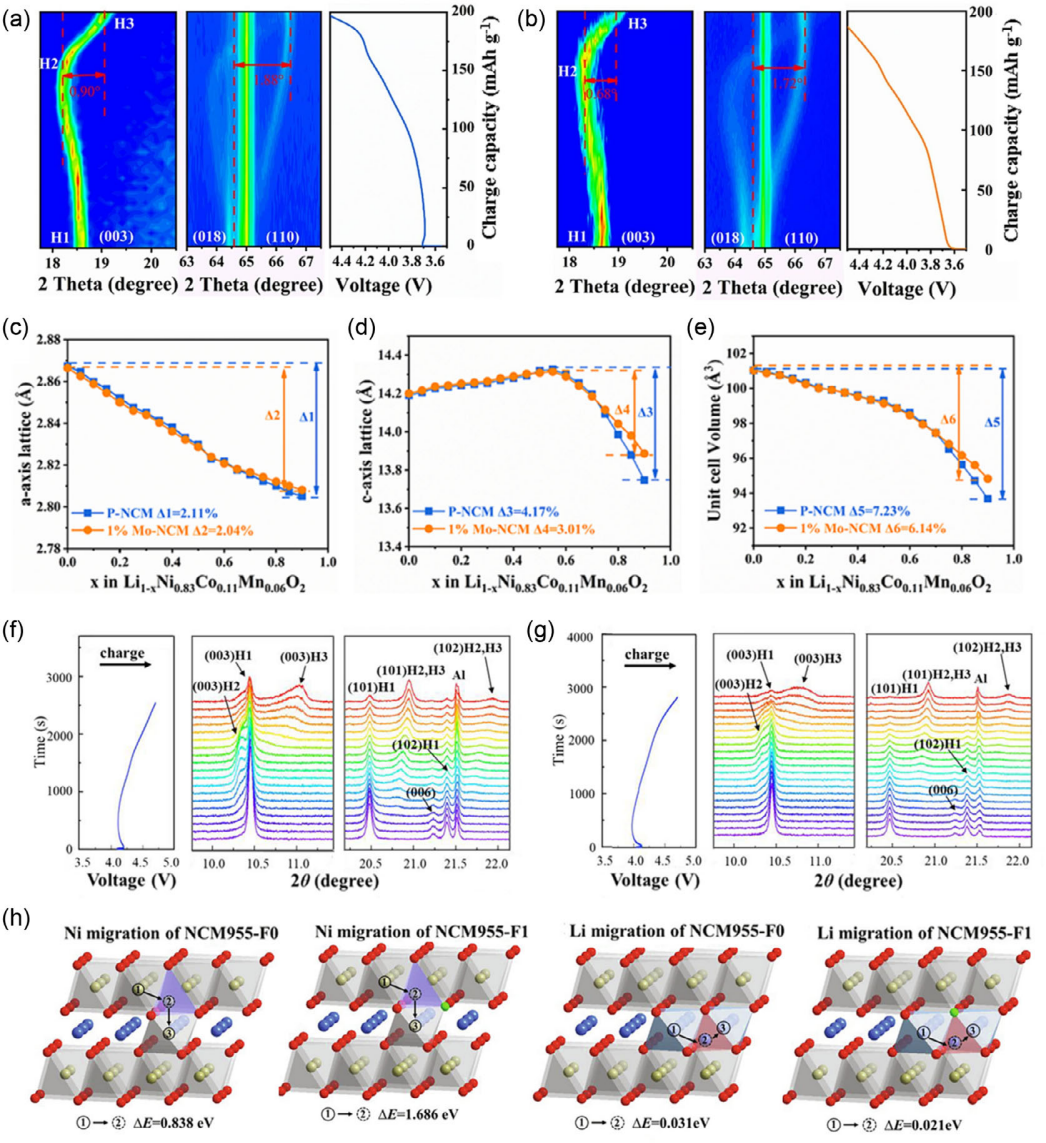
In situ XRD patterns and corresponding charge curves of a) P‐NCM and b) 1% Mo‐NCM (from 3.0 to 4.5 V at 0.2 C). Evolution of c) *c*‐axis and d) *a*‐axis lattice parameters, and e) cell volume during delithiation. Reproduced with permission.^[^ ^153^ ^]^ Copyright 2023, Elsevier. Synchrotron in situ XRD patterns and corresponding charging curves during the initial charging process at a current rate of 1 C for f) NCM955‐F0 and g) NCM955‐F1. h) Structural diagram of the diffusion routes for Ni and Li ions in NCM955‐F0 and NMC955‐F1. Reproduced with permission.^[^ ^154^ ^]^ Copyright 2021, Elsevier.

In situ operando XRD is widely used for the real‐time observation of structural changes in Ni‐rich cathode materials. For instance, Fan et al. conducted in situ XRD analysis using Cu Kα radiation (*λ* ≈ 1.5418 Å) with a scanning range of 15°–75° at a current density of 0.2 C during the initial charge process of LiNi_0.83_Co_0.11_Mn_0.06_O_2_ (P‐NCM) and 1% Mo‐doped LiNi_0.83_Co_0.11_Mn_0.06_O_2_ (1% Mo‐NCM) cathodes.^[^ ^153^ ^]^ This experiment aimed to examine the effect of Mo doping on the structural stability of Ni‐rich cathode materials, as shown in **Figure** **3**a,b, respectively. They found that the lattice parameters *a* and *c*, corresponding to the (110) and (003) Bragg reflections, respectively, gradually shifted upon Li^+^ extraction (Figure 3c,d, respectively). At the end of the charging process at 4.5 V, reductions in the lattice parameters were evident in both the *a* and *c* axes, representing a contraction of the unit cells. Notably, the (003) peak initially shifted toward lower angles, indicating gradual expansion along the *c*‐axis. It then moved toward higher angles, signifying contraction until the end of charging. The change in the *c*‐lattice parameter (Δ*c*) for 1% Mo‐NCM was estimated to be 3.01%, which is significantly less than the 4.17% observed in *P*‐NCM. In contrast, the (110) peak consistently shifted to higher angles throughout the charging process, indicating a continuous decrease in the *a*‐lattice parameter. The change in the *a*‐parameter (Δ*a*) in 1% Mo‐NCM was 2.04%, closely matching that of P‐NCM (2.11%). As a result, the change in unit cell volume (Δ*V*) for 1% Mo‐NCM (6.14%) was substantially lower than that of P‐NCM (7.23%), as presented in Figure 3e. Note that continuous Li^+^ removal weakens the structural framework because charge transfer from O 2*p* to Ni *e*_g_ orbitals reduces O—O Coulombic repulsion, leading to contraction of the Li—O layers along the *c*‐axis. In situ XRD investigations confirmed that Mo doping maintained the ordered structure of the framework even in deep delithiation states, preventing the H2–H3 phase transition and reducing the anisotropic strain, thus preserving the structural integrity of the Ni‐rich cathode materials after cycling.

Moreover, Qiu et al. demonstrated that F substitution at an optimal concentration could enhance both the structural and electrochemical stabilities, as evidenced by synchrotron in situ XRD analysis.^[^ ^154^ ^]^ Figure 3f,g shows a series of XRD patterns of the primary diffraction peaks of LiNi_0.9_Co_0.05_Mn_0.05_O_2_ (NCM955‐F0) and Li(Ni_0.9_Co_0.05_Mn_0.05_)O_1.99_F_0.01_ (NCM955‐F1) during charging to 4.7 V at 1 C, respectively, demonstrating a typical phase transition in Ni‐rich cathode materials. The samples were measured at the synchrotron radiation facility using a sagittal double‐crystal Si(111) monochromator. As Li^+^ was continuously extracted, phase transitions from H1 to H2, and ultimately to H3, were evident. Notably, the intensities of the residual (003)_H1_ and (104)_H1_ peaks of NCM955‐F1 were considerably lower than those of NCM955‐F0 in the fully charged state at 4.7 V. This revealed that F substitution was able to reduce polarization by facilitating complete electrochemical reactions in NCM955‐F1, correlating with its higher capacities at high current densities. Furthermore, the intensity and rightward shift of the (003)_H3_ peak of NCM955‐F1 were significantly less pronounced than those of NCM955‐F0 at the end of charging (4.5–4.7 V). This also suggests that F substitution mitigates the irreversible phase transition from H2 to H3 and reduces internal stress caused by volumetric changes in the unit cell, thereby enhancing structural stability and electrochemical performance. Density functional theory (DFT) calculations were conducted to investigate the energy barriers for the diffusion of Li^+^ and Ni^2+^ in both NCM955‐F1 and NCM955‐F0. The diffusion paths for both Li^+^ and Ni^2+^ were modeled as a transition from an initial octahedral site (site 1) to an intermediate tetrahedral site (site 2), and finally to another octahedral site (site 3), as shown in Figure 3h. The energy barriers for atomic diffusion were carefully calculated based on the energy differences between the sites. The results showed that the energy barrier for Ni^2+^ diffusion in F‐doped NCM955‐F1 increased from 0.838 to 1.686 eV, indicating a more challenging Ni^2+^ migration process with decreased cation mixing. In contrast, the energy barrier for Li^+^ diffusion in NCM955‐F1 was found to be 0.021 eV, which is lower than the 0.031 eV in NCM955‐F0. This reduction confirms that 1% F substitution improves Li^+^ mobility within the Li layers, which is consistent with the enhanced kinetic properties and rate capability observed for NCM955‐F1.

### 3.2 In Situ Operando Raman Techniques

**Figure 4:**
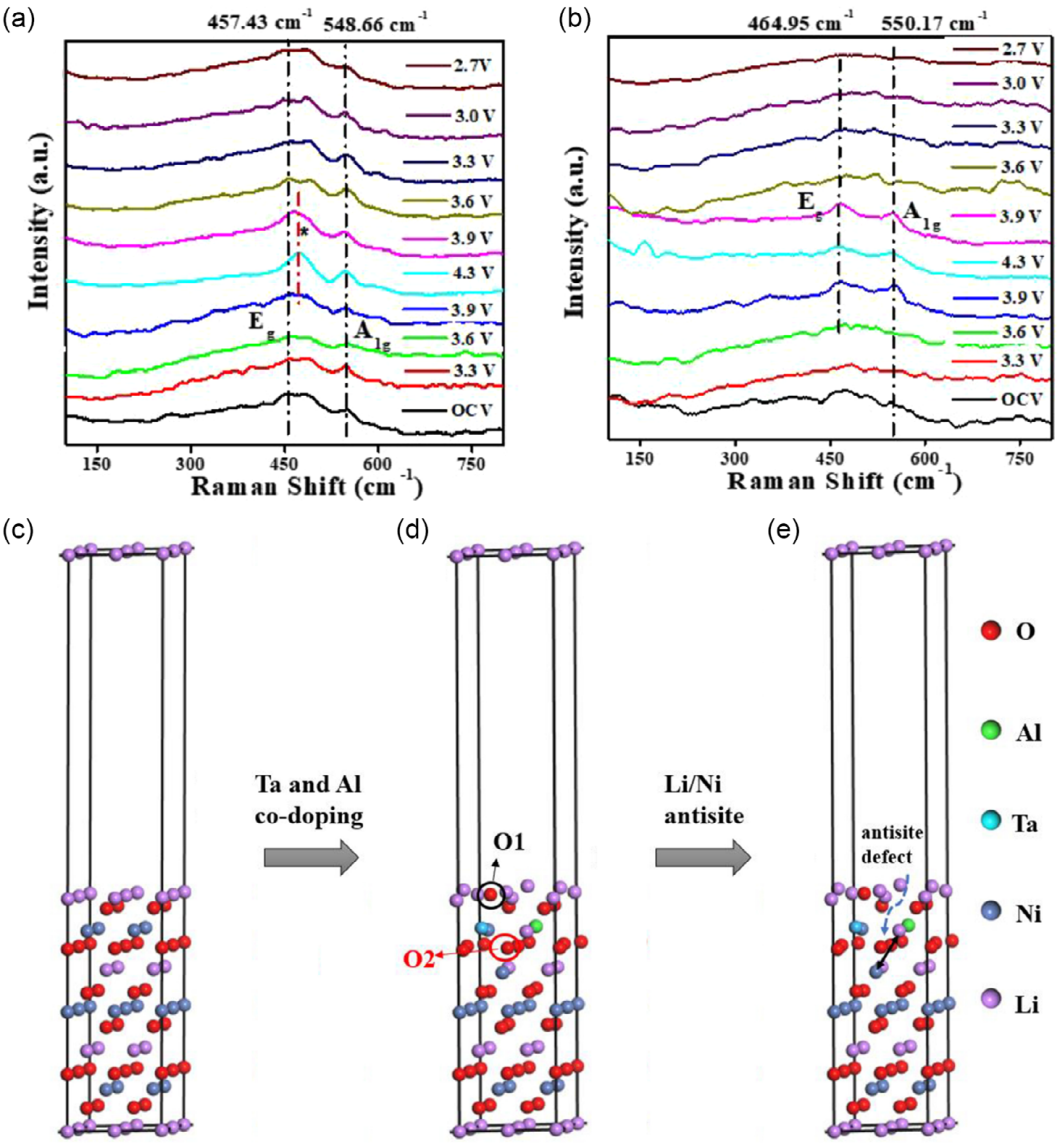
In situ Raman of a) NCM and b) NCMTA at different charge/discharge state. Structural models to examine the surface stability before/after codoping and with/without antisite defect: c) Li_12_Ni_12_O_24_ along with (003) surface, d) Li_12_Ni_12_O_24_ after Ta and Al doping with marked O1 (surface) and O2 (subsurface), and e) Li_12_Ni_12_O_24_ after Ta and Al doping and with one Li/Ni antisite defect formed near the doping site. Reproduced with permission.^[^ ^155^ ^]^ Copyright 2022, Elsevier.

To clarify the relationship between elemental doping and stabilization of the layered structure, Jamil et al. employed in situ Raman spectroscopy, as illustrated in **Figure** **4**a,b.^[^ ^155^ ^]^ At open‐circuit voltage (OCV), two characteristic broadened peaks, *E*_g_ (M—O vibration) and *A*_1g_ (O—M—O bending), appeared around 460 and 550 cm^−1^, respectively. The broadbands observed in LiNi_0.94_Co_0.03_Mn_0.03_O_2_ (NCM) and 0.1 mol% Ta/Al codoped NCM (NCMTA) were attributed to the vibrations of Ni—O (474, 554 cm^−1^), Mn—O (594 cm^−1^), and Co—O (486, 596 cm^−1^), corresponding to the transition metals in the layered R‐3*m* structure. Upon charging the cell from 3.3 V, the *E*_g_ and *A*_1g_ peaks of the NCM exhibited noticeable changes in position and relative intensity. At 3.3 V, a peak at 600 cm^−1^ emerged, similar to the *A*_1g_ mode of Ni—O, resulting from oxygen vibrations toward vacant Li sites, which disappeared at the end of discharge (2.7 V). During the charging process, the intensity of both *E*_g_ and *A*_1g_ bands increased, and the peaks disappeared upon lithiation with no additional features. The intense redox activity at 4.3 V during charging was linked to oxygen release (O^2−^ → O^−^/O) and the reduction of M—O states. In comparison, a broadband for NCMTA at OCV split into two peaks (465.0 and 550.2 cm^−1^) at the end of charge (4.3 V). During lithiation, the growth of this band in NCMTA was less pronounced than in NCM, indicating reduced oxygen loss and smaller changes in lattice parameters. Moreover, no peak at 600 cm^−1^ was observed for NCMTA at the end of discharge (2.7 V), indicating the absence of Li‐deficient MO_6_ octahedron formation.

To further explore the effects of Ta/Al codoping during cycling, transmission electron microscopy (TEM) analyses were performed on both NCM and NCMTA after 100 cycles (Figure S2, Supporting Information). The high‐resolution TEM images of NCM (Figure S2a, Supporting Information) revealed two distinct regions (sites I and II). Region I exhibited voids and confirmed the rock‐salt phase with a selected area electron diffraction pattern assigned to oxygen loss and transition metal dissolution due to side reactions. Region II demonstrated the layered R‐3*m* structure. This observation suggests the infiltration of rock‐salt impurities from the surface toward the bulk of the cathode particles during cycling, leading to phase transitions and electrolyte corrosion. Such processes induce the formation of microcracks induced by anisotropic volume changes, undermining the mechanical stability of the Ni‐rich cathode material and promoting electrolyte infiltration into the particle core, thus expediting surface degradation and internal structural changes of the primary particles. Conversely, the HR‐TEM images of NCMTA (Figure S2b, Supporting Information) showed a well‐ordered layered structure that maintained structural integrity even after 100 cycles.

To explain the stability of the surface structure in detail, first principle calculations were performed. Two oxygen atoms, O1 and O2, were selected to investigate oxygen retention in the structure. The formation energies of oxygen vacancies (*E*_f_) and the binding energies between the oxygen atoms and the matrix surface (*E*_b_) were calculated for three different (003) surface models for 1) pure Li_12_Ni_12_O_24_, 2) Li_12_Ni_12_O_24_ with Ta and Al codoping in the subsurface Ni layer, and 3) Li_12_Ni_12_O_24_ after Ta and Al doping with one Li/Ni antisite defect near the doping site, as displayed in Figure 4c–e, respectively. The calculation results, summarized in Table S2, Supporting Information, indicate that the formation energy increases with Ta and Al doping, with a further increment observed when a Li/Ni antisite defect is present for both O1 and O2. The increased formation energy suggests that creating a Li/Ni antisite defect further enhances surface oxygen stability and prevents the formation of active oxygen species. Furthermore, the binding energy (*E*_b_) with one Li/Ni antisite around Ta and Al (Figure 4f) significantly increases, indicating that oxygen atoms are more difficult to dislocate from the surface after Ta/Al codoping. The superior binding force of Ta—O and Al—O bonds stabilizes the O^2−^ anion framework, hindering oxygen loss from the structure. Moreover, the strong covalency of Ta—O and Al—O bonds provides extra electrons to the oxygen atoms, resulting in higher electron accumulation around Ta and Al compared to Ni. This stabilizes the lattice oxygen during cycling. Oxygen retention in the layered structure mitigates phase transitions to spinel/rock‐salt phases. Hence, the stabilized layered structure enhances structural stability, resulting in significantly improved electrochemical performance and thermal stability.

### 3.3 In Situ Operando XAS

**Figure 5:**
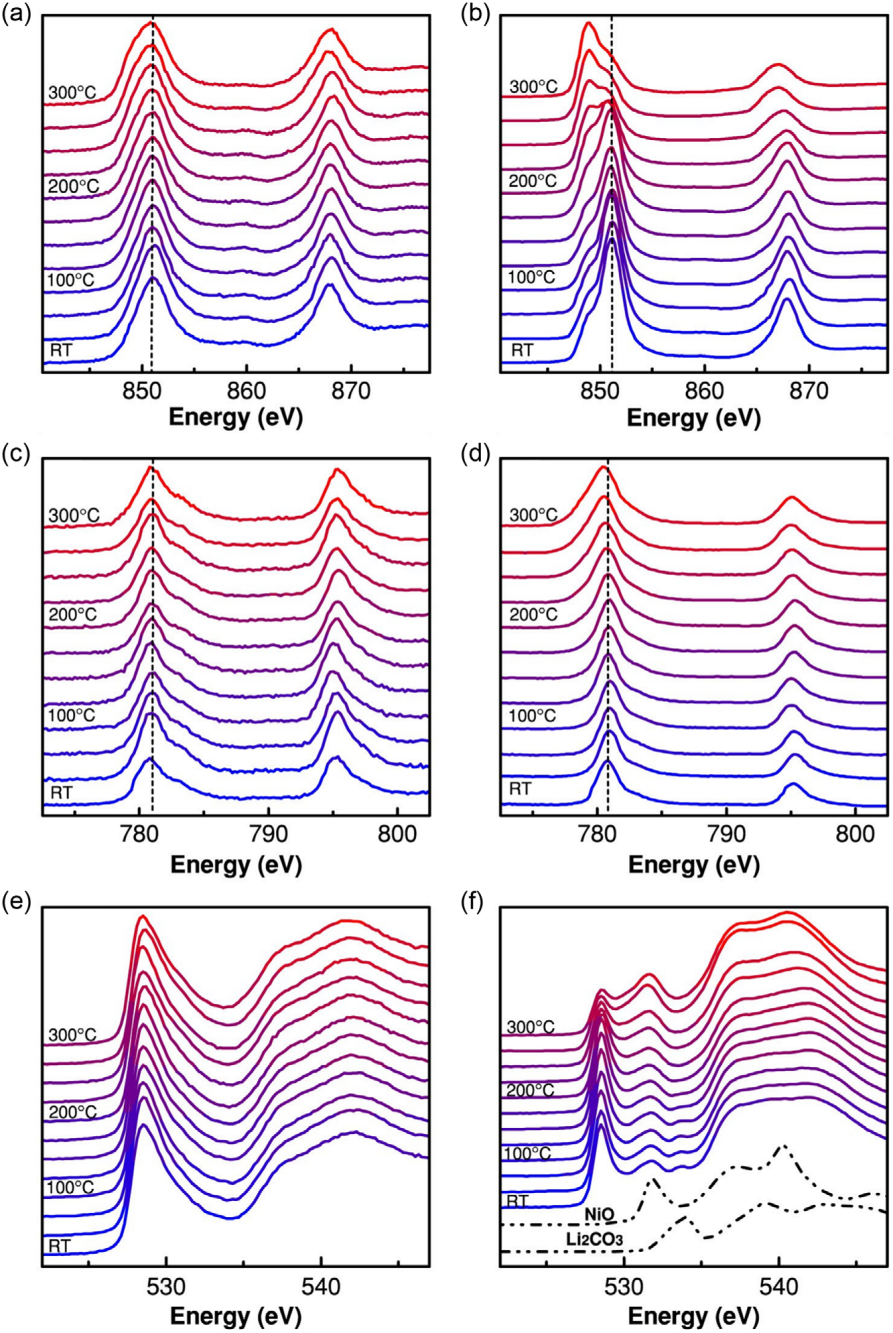
Normalized soft XAS spectra of Li_0.33_Ni_0.8_Co_0.15_Al_0.05_O_2_ cathode material at different temperatures using a) Ni L‐edge FY mode, b) Ni L‐edge PEY mode, c) Co L‐edge FY mode, and d) Co L‐edge PEY mode. Normalized O K‐edge XAS spectra of Li_0.33_Ni_0.8_Co_0.15_Al_0.05_O_2_ cathode material at different temperatures using e) FY mode and f) PEY mode. Reproduced with permission.^[^ ^156^ ^]^ Copyright 2014, Springer Nature.

The normalized Ni L‐edge spectra of Li_0.33_Ni_0.8_Co_0.15_Al_0.05_O_2_ cathode materials were analyzed at various temperatures using both the fluorescent yield (FY) and partial electron yield (PEY) modes, as shown in **Figure** **5**a,b, respectively.^[^ ^156^ ^]^ These absorption spectra, influenced by the spin–orbit interaction of the core hole, distinctly split into two major energy bands: Ni 2*p*_3/2_ (L_3_ edge) and Ni 2*p*_1/2_ (L_2_ edge). These bands offer critical insights into the valence and spin states of Ni^*x*+^, which are characterized by their specific shapes, energy positions, and branching ratios. Analyses conducted in bulk‐sensitive FY mode revealed no shifts in the energy positions of the Ni L_3_ and L_2_ spectra. This observation aligns with the theoretical prediction that the transition to the Fd 3*m* structure does not induce a change in the valence state, precluding any expected shift in the energy positions. Conversely, the surface‐sensitive PEY mode displayed a notable shift to lower energy levels, particularly evident at ≈200 °C. This shift indicated the formation of an NiO‐type rock‐salt structure on the surface of Ni‐rich cathode materials at elevated temperatures. Further investigations were conducted on the normalized Co L‐edge XAS spectra at various temperatures using both the FY and PEY modes, as illustrated in Figure 5c,d. These investigations revealed no energy shifts in the electron yield spectra of the Co species. This lack of spectral change in both the FY and PEY modes suggested the superior thermal stability of Co^*x*+^ compared to Ni^*x*+^. The enhanced thermal stability of the cathode material can be attributed to the partial substitution of Ni with Co.

The study also included an analysis of the normalized O K‐edge XAS spectra of Li_0.33_Ni_0.8_Co_0.15_Al_0.05_O_2_ at different temperatures, utilizing the FY and PEY modes, as shown in Figure 5e,f. The O K‐edge spectra featured a pronounced absorption peak at 528.5 eV, indicating the transition from the O 1*s* orbital to a hybridized state involving the metal 3*d* and O 2p orbitals. Although the FY mode spectra remained largely unchanged, the PEY mode spectra exhibited a significant reduction in intensity of the 528.5 eV peak at temperatures above 200 °C, alongside other spectral variations. Notably, the peak at ≈534 eV diminished, whereas the peak at ≈532 eV became more pronounced with increasing temperature. These changes suggest the decomposition of surface carbonate and formation of reduced divalent Ni^2+^, similar to the rock‐salt NiO structure. These findings from the O K‐edge spectra corroborated the observations from the Ni L‐edge analysis, indicating the onset of thermal reduction reactions at the Ni sites on the surface, leading to the formation of an NiO‐type rock‐salt structure and the consequent release of O at higher temperatures.

**Figure 6:**
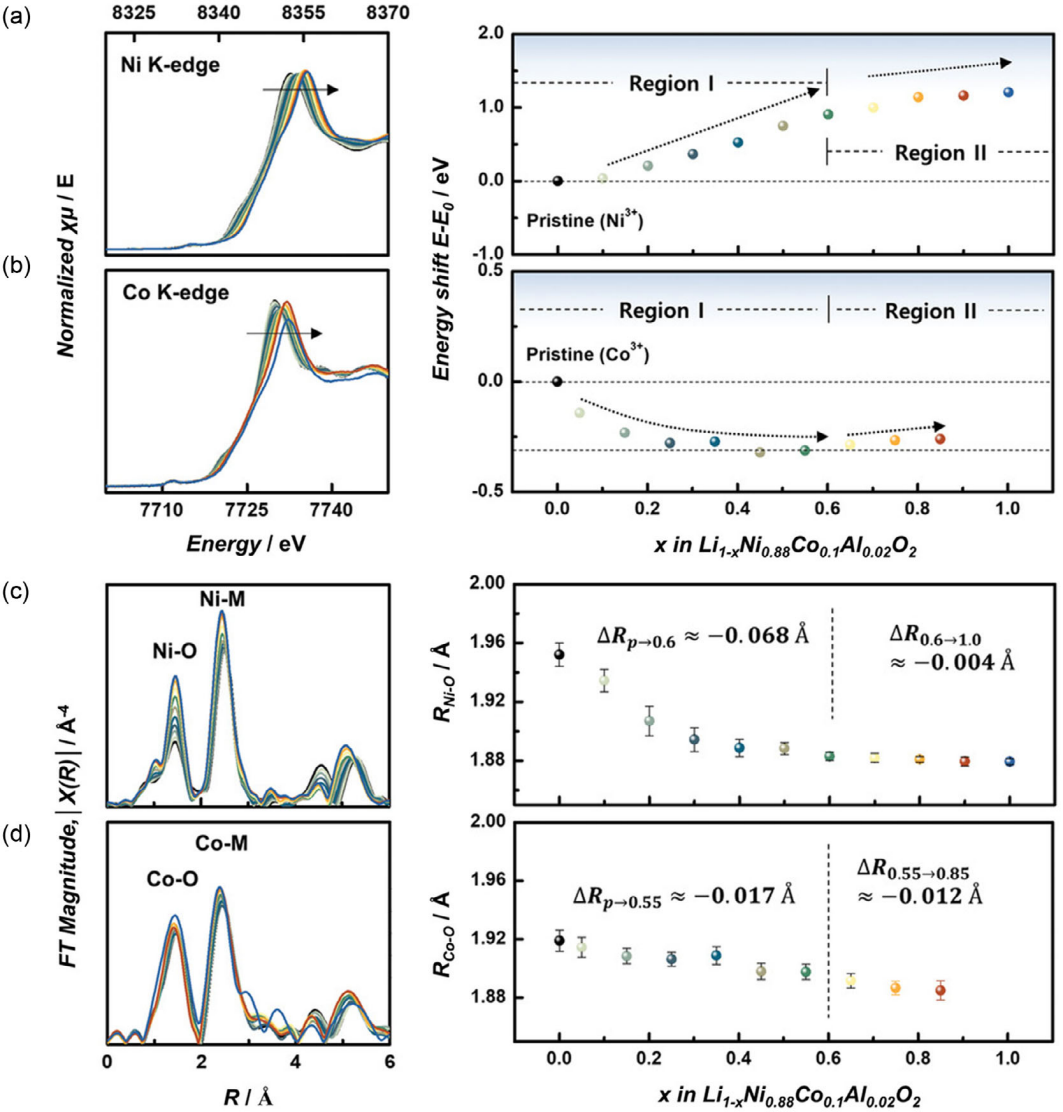
In situ hard XAS. a) Normalized Ni K‐edge and b) Co K‐edge XANES spectra of the Li_1−*x*_Ni_0.88_Co_0.1_Al_0.02_O_2_ (NCA) cathode material during de‐intercalation process, and the corresponding energy shift *E−E*_0_ of the Ni and Co K‐edge at the half‐height of the edge step is plotted to the right of each XANES spectra. The *k* ^3^‐weighted Fourier transform magnitudes of the c) Ni K‐edge and d) Co K‐edge EXAFS spectra of NCA cathode material during the deintercalation process, and the corresponding structural parameters of the first coordination shell Ni–O and Co–O are plotted to the right of EXAFS spectra. Reproduced with permission.^[^ ^157^ ^]^ Copyright 2020, Wiley.

Through in situ hard XAS, insights into the charge compensation mechanism and structural behavior of Ni‐rich materials can be obtained. **Figure** **6**a,b displays the normalized Ni and Co K‐edge XANES spectra of the Li_1−*x*_Ni_0.88_Co_0.1_Al_0.02_O_2_ (L_1−*x*_NCA) cathode material during the charging process, focusing on the energy shifts (*E*−*E*_0_) at both metal K‐edges.^[^ ^157^ ^]^ The Ni K‐edge XANES spectra exhibited a consistent shift to higher energies during Li^+^ extraction, indicating the oxidation of Ni^*x*+^. This oxidation is evidenced by a gradual increase in the average oxidation state of Ni^*x*+^, with a noticeable edge position change occurring at the *x* = 0.6 deintercalation level in L_1−*x*_NCA (Figure 6a). In contrast, the Co K‐edge XANES spectra showed no distinct edge shifts, making it challenging to determine the oxidation state of Co^*x*+^. The Co 1s to 3 d or 4p electronic dipole transition was influenced by the Co‐O local environment, which was further complicated by significant changes in the Ni—O coordination and rearrangement of Li^+^ within the Li layers. This complexity in the Co K‐edge XANES spectra is common in Ni‐rich cathode materials containing Co, where Co is often considered a spectator ion, particularly at voltages up to ≈4.6 V versus Li/Li^+^ (Figure 6b). EXAFS spectra revealed that the first Ni—O coordination peaks intensified during the charging process. This indicated the oxidation of Ni^3+^ to Ni^4+^ and the transition from a distorted NiO_6_ octahedron to a symmetric one, characterized by shorter and identical Ni—O bonds (1.879 Å). The second Ni—M (M = Ni, Co) coordination peaks also showed increased intensity due to the reorganization of the NiO_6_ octahedra upon the oxidation of Ni^*x*+^ (Figure 6c). In the Co K‐edge EXAFS spectra, the first coordination peaks remained mostly unchanged, but the intensities of the second Co—M coordination peaks increased owing to the reordering of the surrounding NiO_6_ octahedra. Quantitative analysis of the EXAFS spectra provided structural parameters for the first and second coordination shells around Ni and Co. In region I (0.0 ≤ *x* ≤ 0.6) of the charge process, significant alterations occurred in the first coordination peaks, with the Ni—O bond length decreasing sharply by 0.069 Å, from 1.952 to 1.883 Å. This change, together with the known ionic radius difference between Ni^3+^ and Ni^4+^ (0.08 Å), strongly suggested the oxidation of Ni^*x*+^ within this region. After *x* = 0.6, in region II (0.6 ≤ *x* ≤ 1.0), the Ni—O bond length exhibited a linear and slight reduction, indicating a deceleration in the reaction rate of Ni sites. The Co K‐edge EXAFS analysis showed a linear decrease in the Co—O bond length up to *x* = 0.85, with minimal changes in the first coordination peaks, highlighting the complex behavior of Co^*x*+^ during Li^+^ extraction (Figure 6d). These XANES and EXAFS analyses for Ni and Co collectively revealed that as more than 0.6 mol of Li^+^ was extracted, the reaction rates of the transition metals were reduced, with hole generation in O^2−^.

### 3.4 In Situ Operando DEMS Techniques

**Figure 7:**
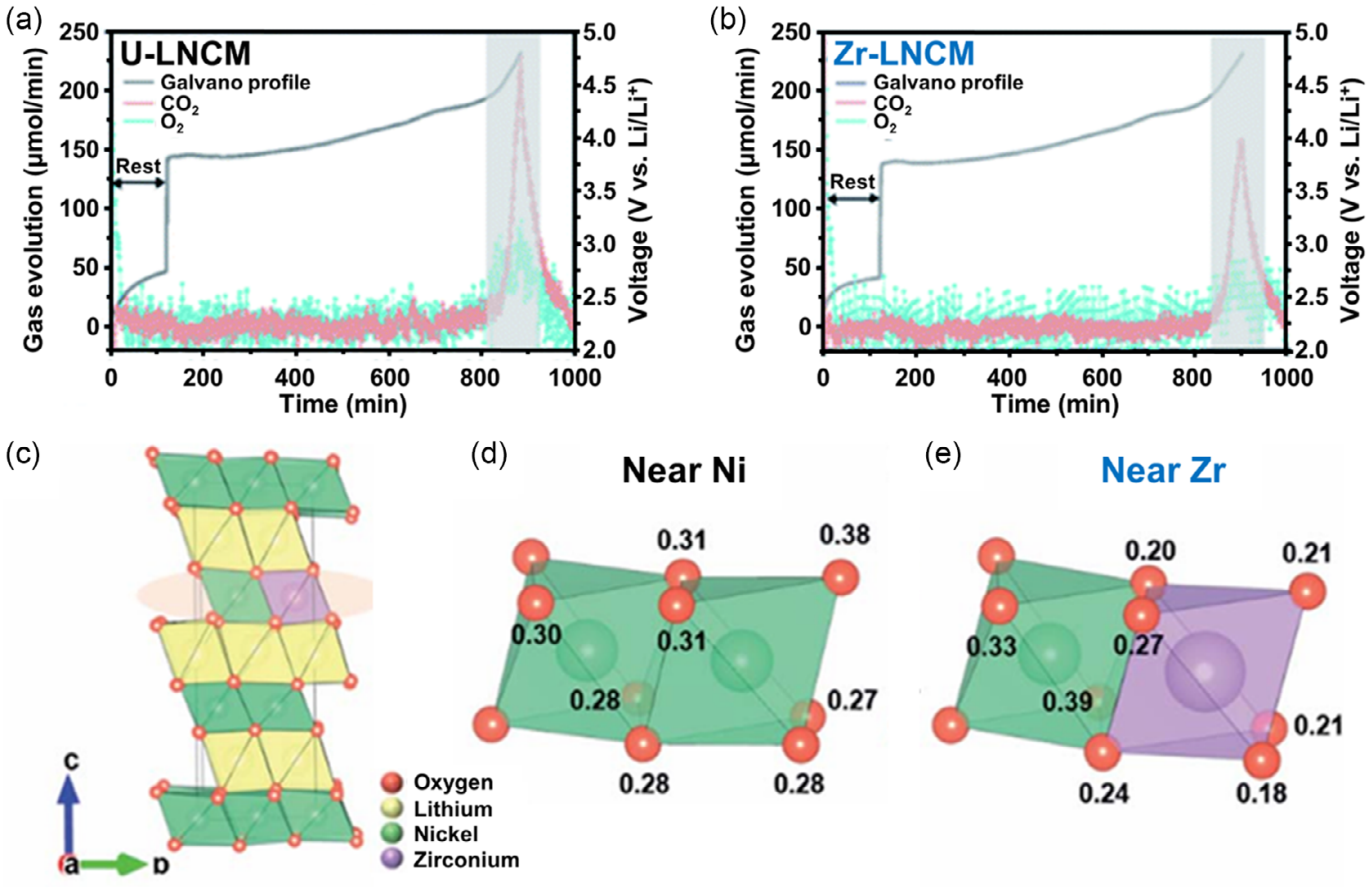
DEMS spectra representing a) U‐LNCM and b) Zr‐LNCM when charged to 4.8 V at 0.1 C current density. c) Atomic structure of Li_12_Ni_11_ZrO_24_, d) atomic structure near Ni atom, and e) atomic structure near Zr atom. The number on (d,e) indicates the charge loss of corresponding oxygen when delithiated from Li_12_Ni_11_ZrO_24_ to Li_2_Ni_11_ZrO_24_, which is obtained by the Bader charge analysis. Reproduced with permission.^[^ ^158^ ^]^ Copyright 2021, The Royal Society of Chemistry.

Gas generation within LIBs is recognized as an adverse occurrence due to its contribution to increased pressure and temperature. Monitoring gas evolution is particularly important for developing highly reliable LIBs with enhanced safety and an extended cycle life. In this context, Jung et al. employed operando DEMS to investigate the advantageous effects of Zr doping on NCM, particularly in assessing the rate of gas evolution during the charging process.^[^ ^158^ ^]^ The study observed that O_2_ was released following the H2 phase for both cathode materials, with a notable intensification in gas evolution as the charging potential increased, as shown in **Figure** **7**a. This acceleration in gas production was attributed to the destabilization of undoped LiNi_0.92_Co_0.04_Mn_0.04_O_2_ (U‐LNCM) at elevated delithiation states. Moreover, the simultaneous evolution of CO_2_ gas, stemming from the electrolyte decomposition induced by singlet oxygen released from U‐LNCM, was recorded, and its rate also increased with higher charging voltages. Notably, Zr doping (Zr‐LNCM) was found to delay the initiation of gas evolution and decrease the rate of O_2_ gas release, as shown in Figure 7b. Furthermore, the generation of CO_2_ gas was reduced, indicating that Zr doping effectively mitigated the chemical oxidation of the electrolyte, as shown in Figure 7b. Therefore, the operando DEMS results conclusively demonstrated that Zr doping in LNCM significantly curbed the release of O_2_ and the decomposition of the electrolyte, thereby contributing to the enhanced stability of Ni‐rich cathode materials. To understand the fundamental impact of Zr doping on O_2_ evolution, first‐principles calculations were conducted using the projector‐augmented wave method. The variation in doping concentration between the computational models and experimental setups was deemed inconsequential because the primary objective of these calculations was to ascertain the potential influence of Zr on O charge depletion at high states of charge (SOC). The study examined the evolution of O charge density by altering the Li content within the Li_1−*x*_NiO_2_ framework, comparing the O charge density adjacent to Zr atoms against that of O atoms situated farther from Zr. In Figure 7d,e, the numerical values adjacent to the O atoms denote the extent of charge loss in O during Li^+^ extraction. At a deep charge level (*x* = 0.84), O atoms exhibited a deficiency in SOC relative to their pristine state at *x* = 0, corroborating the findings of previous studies.^[^ ^159^, ^160^ ^]^ Notably, the computational results revealed that the O atoms in close proximity to Zr (Figure 7e) experienced a lower degree of charge density loss than those distant from Zr. These DFT calculation outcomes aligned with experimental observations, affirming that Zr doping effectively mitigated the charge loss of O in deep charging states.

**Figure 8:**
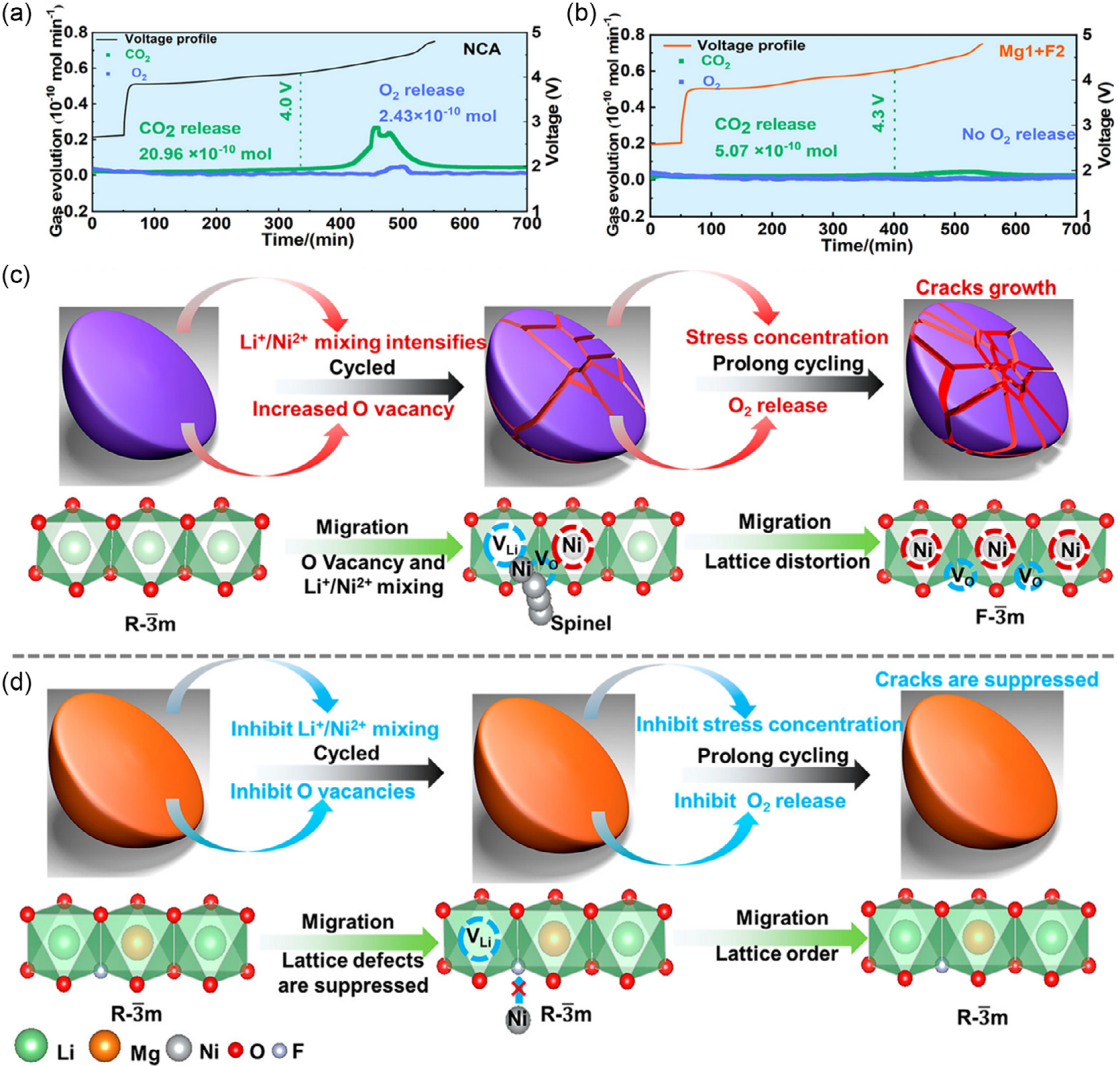
In situ DEMS measurements for a) NCA and b) Mg1 + F2. Schematic diagram of the structural evolution of c) NCA and d) Mg1 + F2. Reproduced with permission.^[^ ^161^ ^]^ Copyright 2023, American Chemical Society.

To elucidate the effects of cation and anion codoping, Zhou et al. performed in situ DEMS analysis on LiNi_0.8_Co_0.15_Al_0.015_O_2_ (NCA) and Mg‐ and F‐doped NCA (Mg1 + F2) during their initial charging process, as illustrated in **Figure** **8**a,b.^[^ ^161^ ^]^ NCA cathodes exhibited more significant CO_2_ emissions at 4.0 V than their Mg1 + F2 counterparts did at 4.3 V, indicating the inherent instability of lattice O in NCA. This instability led to adverse side reactions in the electrolyte, resulting in CO_2_ production. Importantly, upon charging to 4.4 V, O_2_ evolution was observed in NCA, a phenomenon absent in Mg1 + F2 cathode materials. This release of gas, particularly after prolonged cycling, could cause severe structural damage, including microcracks, and poses substantial safety risks in practical applications. The absence of O_2_ in the Mg1 + F2 cathode materials indicated the effectiveness of Mg^2+^ and F^−^ codoping in stabilizing lattice O to prevent O_2_ release. The schematic diagrams in Figure 8c,d illustrate the mechanisms behind the performance enhancements attributed to Mg and F codoping. In NCA, the formation of Li vacancies led to Ni^2+^ migration toward adjacent tetrahedral sites, initially forming a spinel structure, and subsequently toward Li vacancies, causing Li^+^/Ni^2+^ cation mixing. High cutoff voltages (≥4.5 V) exacerbated this situation by creating O vacancies to maintain the charge balance, which, in turn, facilitated O_2_ evolution and further promoted Li^+^/Ni^2+^ cation mixing. This sequence of events amplified the lattice distortion and stress concentration, ultimately leading to microcrack propagation. The origin of such anisotropic stress concentrations is related to the interaction between O vacancies and Li^+^/Ni^2+^ mixing, where Ni^2+^ migration to the Li layer not only formed Ni—O phases but also introduced dislocations within the layered structure, increasing stress concentrations and, consequently, material degradation. Conversely, in the Mg1 + F2 cathode materials, Mg^2+^ and F^−^ codoping increased the energy barriers for Ni^2+^ migration and O escape, respectively. This adjustment effectively inhibited the formation of the spinel phase and O_2_ release, thereby reducing the stress concentration and significantly limiting microcrack formation. This comparative analysis highlighted the crucial role of strategic elemental codoping in improving the stability and safety of Ni‐rich cathode materials.

## 4 Conclusion and Perspectives

The development and enhancement of advanced Ni‐rich cathode materials, particularly through elemental doping and substitution techniques, require a thorough understanding of the complex phase transitions and key degradation mechanisms during cycling. This knowledge is essential for enhancing the performance, durability, and lifespan of the cathode materials in high‐energy LIBs for emerging applications. By conducting comprehensive analyses of how these materials respond to various operational conditions, researchers can identify the crucial factors that influence their stability and efficiency. This integrated approach facilitates the tailored optimization of Ni‐rich cathode materials to meet the growing demand for high‐energy LIBs in modern society. Furthermore, it is crucial to clarify the reaction mechanisms affected by elemental doping and substitution in Ni‐rich cathode materials to advance this area. This deeper understanding is pivotal for engineering LIBs that are not only more efficient but also have extended service lives, as this plays a significant role in fine‐tuning the material properties of Ni‐rich cathode materials for high‐energy LIBs.

**Figure 9:**
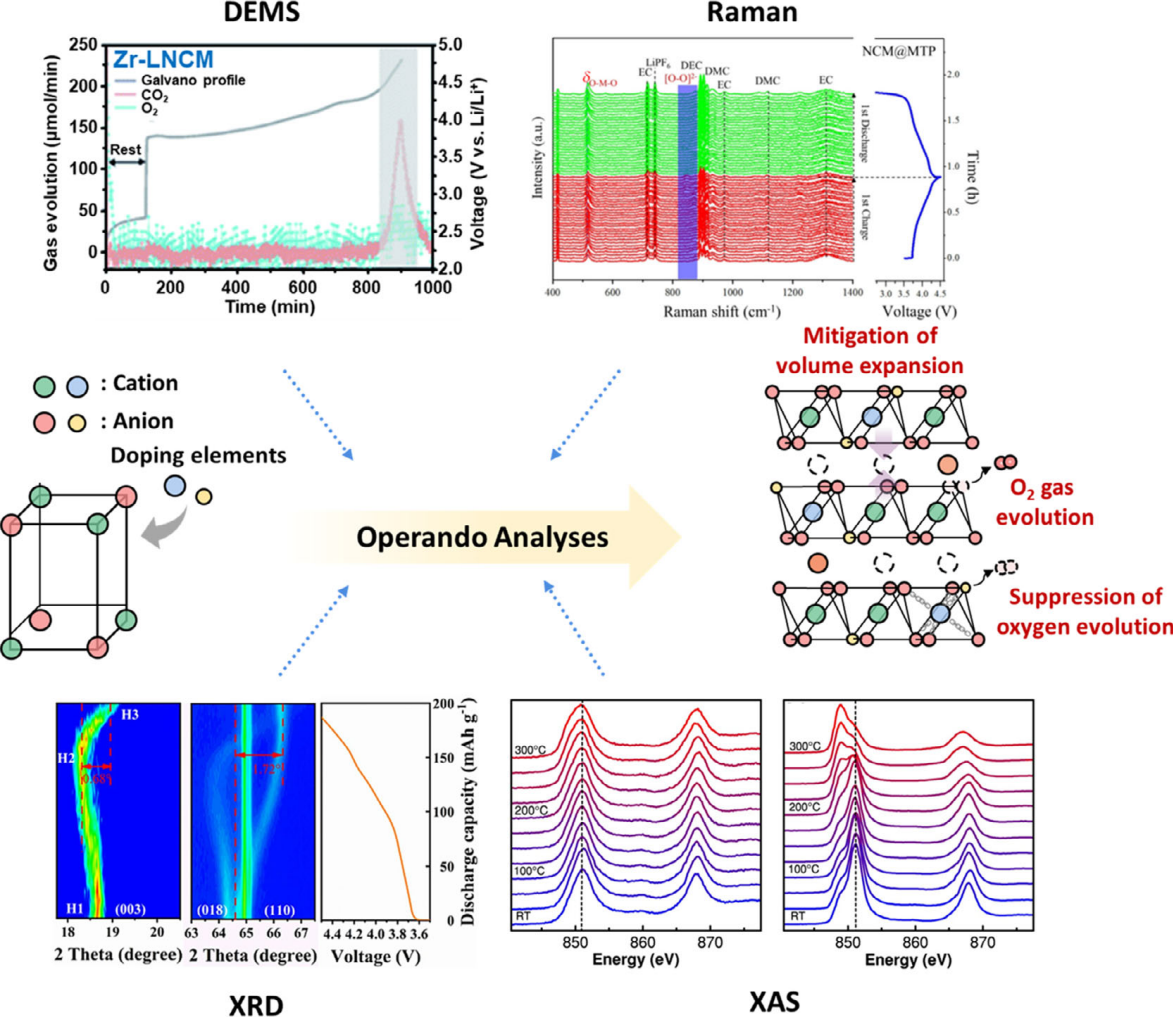
Schematic illustration of the cathode material stabilization mechanism elucidated through in situ operando analyses.

In this article, we reviewed and discussed the pivotal role of in situ operando analytical techniques in elucidating the dynamic reaction mechanisms within Ni‐rich cathode systems during electrochemical processes. Specifically, we highlighted the in‐depth insights provided by XRD, Raman spectroscopy, XAS, and DEMS. Each of these methodologies reveals distinct facets of material behavior under operational stresses, thereby enriching our understanding of the structural evolution, phase transitions, and release of gaseous species in Ni‐rich cathode materials during cycling. In situ operando XRD is a powerful technique that offers unparalleled resolution for tracking structural modifications and phase transitions in real time, thus providing valuable insights into the fundamental changes that underpin electrochemical performance. In situ operando Raman spectroscopy, which is sensitive to vibrational modes, complements this by mapping molecular and crystal lattice vibrations, thereby revealing subtle structural adjustments and chemical heterogeneities. In situ operando XAS presents a detailed view of the electronic structure and coordination chemistry around specific atoms, thus offering a microscopic view of the electronic and structural rearrangements that accompany phase transitions. Finally, in situ operando DEMS serves as a critical tool for the real‐time monitoring of evolved gaseous species during electrochemical processes, providing key insights into the gaseous byproducts and degradation mechanisms inherent to cycling. Together, these in situ operando analytical methods form a robust framework for dissecting the multifaceted processes occurring within materials under operational conditions, laying the foundation for future innovations and advancements in materials science. These in‐depth analyses demonstrate that the targeted doping and substitution of cations or anions can significantly alter their internal bonding configurations within the crystal structures of Ni‐rich cathode materials. Such alterations provide several advantages: 1) Enhancing the interactions between cations and anions counters the expansion of the lattice observed during Li^+^ extraction to improve the structural and electrochemical stabilities of Ni‐rich cathode materials. 2) Reducing O_2_ evolution within Ni‐rich cathode materials limits the incidence of irreversible phase transitions and chemical reactions associated with electrolyte decomposition, extending the cycle life. 3) Modulating the states of cation–anion bonding can decrease the energy barriers for Li^+^ diffusion, thus enhancing the kinetic properties and rate performance of Ni‐rich cathode materials (**Figure** **9**). Careful control of the elemental doping process is crucial to avoid the incorporation of impurity phases, which may adversely affect the electrochemical performance of these cathode materials. Future research should aim to identify economical and effective dopants capable of cosubstituting at the Li, TM, and O sites. This approach is expected to synergistically improve both the electrochemical and thermal properties of Ni‐rich cathode materials, rendering them more suitable for commercial applications.

Thus, the use of in situ operando analysis techniques to investigate the effects of doping or substitution provides a powerful method for elucidating the reaction mechanisms in Ni‐rich cathode materials, which enhance their structural stability and longevity.

This review emphasizes the pivotal role of in situ operando methodologies in advancing our understanding of Ni‐rich cathode materials, particularly in elucidating the doping/substitution mechanisms behind performance improvements of LIBs. By focusing on the Ni‐rich layered cathode material as a representative example, we demonstrate how these advanced techniques offer valuable insights into the physical and chemical changes induced by elemental doping/substitution. The application of the in situ operando techniques can extended beyond Ni‐rich cathodes to a wide range of energy storage materials, making this review a versatile resource for researchers. Through the integration of doping/substitution strategies with state‐of‐the‐art in situ operando analyses, we have highlighted the potential to significantly optimize Ni‐rich cathode materials and enhance the design of high‐energy LIBs.

## Conflict of Interest

The authors declare no conflict of interest.

## Supporting information

Supplementary Material
