# Supplementary material for: Comprehensive Understanding of Elemental Doping and Substitution of Ni‐Rich Cathode Materials for Lithium‐Ion Batteries via In Situ Operando Analyses
Source: Small Sci. 2024 Jul 8;4(10):2400165. doi: 10.1002/smsc.202400165 (PMC11935164; doi:10.1002/smsc.202400165)
Supplement: Supplementary file 1 — Supplementary Material [file SMSC-4-2400165-s001.pdf]

Supporting Information

Comprehensive Understanding on Elemental Doping and Substitution of Ni-rich Cathode Materials for Lithium-Ion Batteries via *In situ operando* Analyses

Yun Seong Byeon[1,†], Wontae Lee[4,†],Dongil Kim[1], Jaewoo Jung[1], Min-Sik Park[1,] and Won-Sub Yoon[2,3,]**

Element	Cathode	Capacity retention	Function	Analysis method	Reference
B	$LiNi_{0.85}Co_{0.10}Mn_{0.05}O_2$	94.0% (3.0-4.3 V @ 1C, 123 cycles)	Inhibition of oxygen release	*ex situ* (TEM, NMR)	[1]
	$LiNi_{0.9}Co_{0.05}Mn_{0.05}O_2$	91.0% (2.7-4.3 V @ 0.5C, 100 cycles)	Enhancing structural integrity	ex situ (SEM, TEM)	[2]
Na	$LiNi_{0.8}Co_{0.15}Al_{0.05}O_2$	81.6% (3.0-4.3 V @ 1C, 300 cycles)	Pillar effect	*ex situ* (SEM, XRD)	[3]
Mg	$LiNi_{0.83}Co_{0.12}Mn_{0.05}O_2$	87.2% (2.8-4.5 V @ 1C, 200 cycles)	Pillar effect	*ex situ* (SEM, EIS)	[4]
	$LiNi_{0.8}Co_{0.05}Mn_{0.15}O_2$	87.9% (2.7-4.4 V @ 1C, 200 cycles)		*ex situ / in situ* (SEM, TEM / XRD)	[5]
Al	$LiNi_{0.8}Co_{0.1}Mn_{0.1}O_2$	78.9% (2.7-4.3 V @ 1C, 200 cycles)	Suppression of cation mixing	ex situ (TEM)	[6]
	$LiNi_{0.92}Co_{0.04}Mn_{0.04}O_2$	86.0% (2.7-4.3 V @ 0.5C, 100 cycles)	Suppression of microcracks	ex situ (SEM, TEM)	[7]

Ti	$LiNi_{0.83}Co_{0.11}Mn_{0.06}O_2$	89.9% (2.8-4.5 V @ 1C, 200 cycles)	Enhancing structural integrity	ex situ (SEM)	[8]
Zn	$LiNi_{0.83}Co_{0.11}Mn_{0.06}O_2$	84.0% (3.0-4.5 V @ 0.2C, 100 cycles)	Facilitate Li^+ diffusion	ex situ (SEM)	[9]
Ga	$LiNi_{0.8}Co_{0.1}Mn_{0.1}O_2$	89.5% (2.8-4.3V @ 1C, 100 cycles)	Inhibition of oxygen release	ex situ (SEM, XRD)	[10]
Zr	$LiNi_{0.8}Co_{0.1}Mn_{0.1}O_2$	71.0% (3.0-4.5 V @ 1C, 200 cycles)	Pillar effect	ex situ / in situ (XPS / XRD)	[11]
Nb	$LiNi_{0.8}Co_{0.1}Mn_{0.1}O_2$	84.0% (2.7-4.6 V @ 5C, 200 cycles)	Suppression of cation mixing	in situ (XRD)	[12]
Mo	$LiNi_{0.83}Co_{0.11}Mn_{0.06}O_2$	98.7% (2.7-4.3 V @ 1C, 200 cycles)	Enhancing structural integrity	ex situ / in situ (XPS / XRD)	[13]
Y	$LiNi_{0.8}Co_{0.1}Mn_{0.1}O_2$	98.4% (2.8-4.5 V @ 1C, 100 cycles)	Facilitate Li^+ diffusion	ex situ (EIS)	[14]
F	$LiNi_{0.8}Co_{0.1}Mn_{0.1}O_2$	92.5 (2.8-4.3 V @ 1C, 100 cycles)	Facilitate Li^+ diffusion	ex situ (EIS)	[15]
	$LiNi_{0.9}Co_{0.05}Mn_{0.05}O_2$	95.5% (2.8-4.3 V @ 2C, 100 cycles)	Suppression of cation mixing	ex situ / in situ (XANES / XRD)	[16]
S	$LiNi_{0.8}Co_{0.15}Mn_{0.05}O_2$	80.4% (2.8-4.3 V @ 1C, 240 cycles)	Facilitate Li^+ diffusion	ex situ (EIS)	[17]
Br	$LiNi_{0.815}Co_{0.15}Al_{0.035}O_2$	73.7% (2.8-4.5 V @ 0.5C, 100 cycles)	Facilitate Li^+ diffusion	ex situ (EIS)	[18]

Table S1. Comparison of doping & substitution elements, capacity retention, function, and analysis method for Ni-rich cathodes.

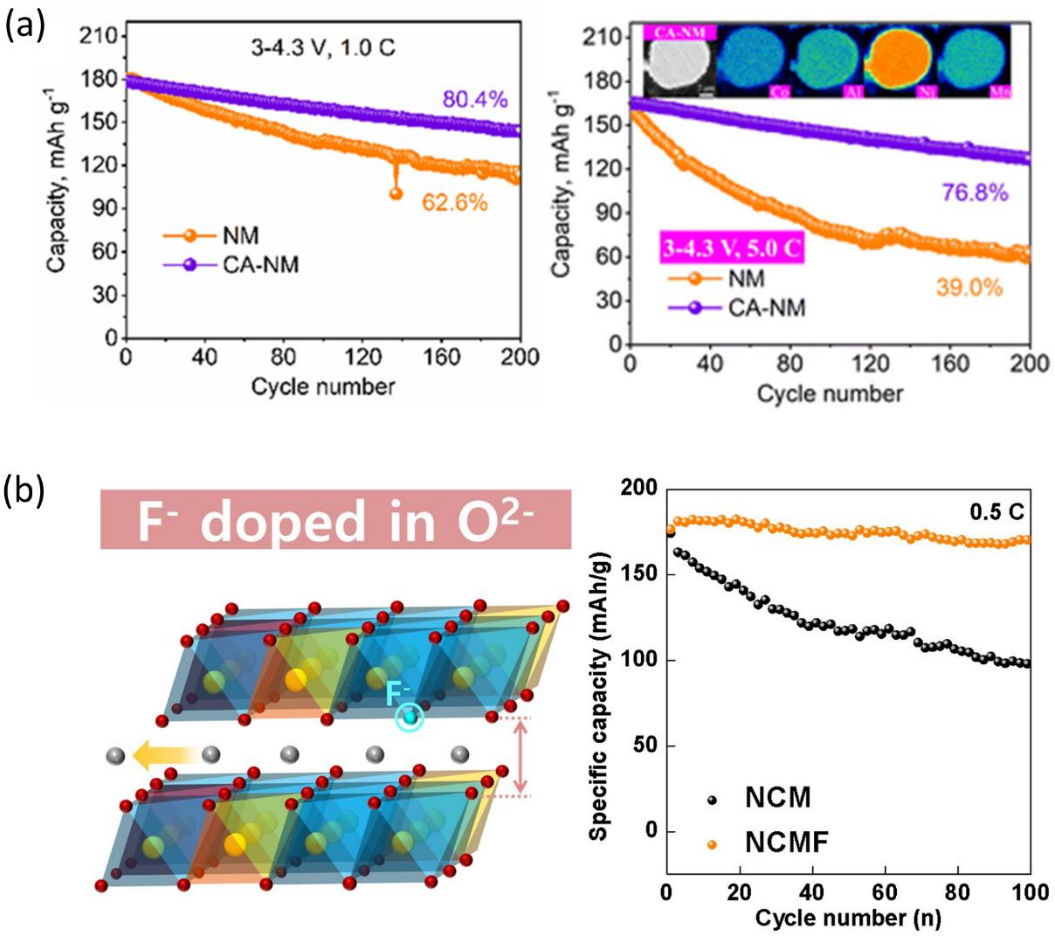

Figure S1. (a) Long-term cycling performance of NM and co-substitution CA-NM. (b) Specific discharge capacities of the NCM and NCMF at a current density of 100 mA g^{-1} in the potential range of 2.8–4.3 V *vs.* Li/Li^{+} for 150 cycles.

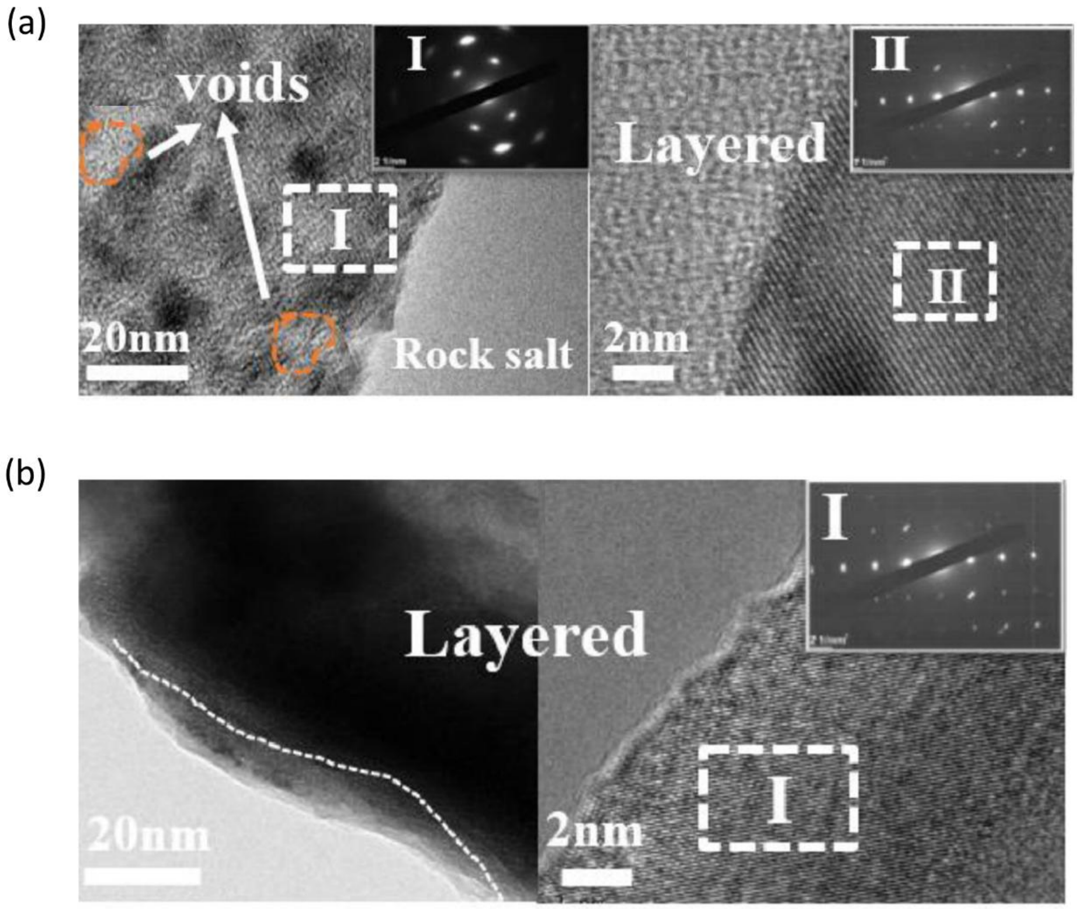

Figure S2. HRTEM along with SAED of cycled electrodes (a) NCM and (b) NCMTA after 100 cycles between 2.7–4.3 V at 0.5 C.

Structural Model	E_f – O1 (eV/atom)	E_f – O2 (eV/atom)	E_b – O1 (eV/atom)	E_b – O2 (eV/atom)
(003) surface in $Li_{12}Ni_{12}O_{24}$	3.05	0.52	7.37	4.84
(003) surface in $Li_{12}Ni_{10}TaAlO_{24}$	4.56	3.36	8.88	7.68
(003) surface in $Li_{12}Ni_{10}TaAlO_{24}$ with antisite defect	4.96	5.19	9.28	9.52

Table S2. The formation energy of surface oxygen (O1) and subsurface oxygen (O2) for (003) surface of $Li_{12}Ni_{12}O_{24}$ and $Li_{12}Ni_{10}TaAlO_{24}$ with one antisite defect.

Reference

[1] C. Roitzheim, L.-Y. Kuo, Y. J. Sohn, M. Finsterbusch, S. Möller, D. Sebold, H. Valencia, M. Meledina, J. Mayer, U. Breuer, P. Kaghazchi, O. Guillon, D. Fattakhova-Rohlfing, *ACS Appl. Energy Mater.* **2022**, *5*, 524.

[2] K.-J. Park, H.-G. Jung, L.-Y. Kuo, P. Kaghazchi, C. S. Yoon, Y.-K. Sun, *Adv. Energy Mater.* **2018**, *8*, 1901202.

[3] Y.-Y. Wang, Y.-Y. Sun, S. Liu, G.-R. Li, X.-P. Gao, *ACS Appl. Energy Mater.* **2018**, *1*, 3881.

[4] Y. Lv, X. Cheng, W. Qiang, B. Huang, *J. Power Sources* **2020**, *450*, 227718.

[5] K. Yang, Y. Yi, C. Yang, F. Liu, K. Wang, J. Cao, Z. Chen, *Chem. Eng. J.* **2023**, *474*, 145554.

[6] Y.-C. Li, W. Xiang, Z.-G. Wu, C.-L. Xu, Y.-D. Xu, Y. Xiao, Z.-G. Wang, C.-J. Wu, G.-P. Lv, X.-D. Guo, *Electrochim. Acta* **2018**, *291*, 84.

[7] U.-H. Kim, S.-B. Lee, J.-H. Ryu, C. S. Yoon, Y.-K. Sun, *J. Power Sources* **2023**, *564*, 232850.

[8] X. Chung, Y. Li, G. Zhao, W. Qiang, B. Huang, *Ceram. Int.* **2022**, *48*, 27849.

[9] L. Wang, J. Qin, Z. Bai, H. Qian, Y. Cao, H. M. K. Sari, Y. Xi, H. Shan, S. Wang, J. Zuo, X. Pu, W. Li, J. Wang, X. Li, *Small Struct.* **2022**, *3*, 2100233.

[10] L. Wu, X. Tang, X. Chen, Z. Rong, W. Dang, Y. Wang, X. Li, L. Huang, Y. Zhang, *J. Power Sources* **2020**, *445*, 227337.

[11] K. Wu, J. Jiao, N. Li, M. Wang, G. Jia, Y. L. Lee, R. Dang, X. Deng, X. Xiao, Z. Wu, *J. Phys. Chem. C* **2021**, *125*, 10260.

[12] Z. Zhao, C. Li, Z. Wen, Z. Yang, S. Lu, X. Zhang, S. Chen, B. Wu, F. Wu, D. Mu, *Chem. Eng. J.* **2023**, *461*, 142093.

[13] F. Fan, R. Zheng, T. Zeng, H. Xu, X. Wen, X. Wang, G. Tian, S. Wang, C. Zeng, W. Xiang, C. Shu, *Chem. Eng. J.* **2023**, *477*, 147181.

[14] M. Zhang, H. Zhao, M. Tan, J. Liu, Y. Hu, S. Liu, X. Shu, H. Li, Q. Ran, J. Cai, X. Liu, *J. Alloys Compd.* **2019**, *774*, 82.

[15] P. Zhang, Z. Liu, B. Ma, P. Li, Y. Zhou, X. Tain, *Ceram. Int.* **2021**, *47*, 33843.

[16] Q.-Q. Qiu, S.-S. Yuan, J. Bao, Q.-C. Wang, X.-Y. Yue, X.-L. Li, X.-J. Wu, Y.-N. Zhou, *J. Energy Chem.* **2021**, *61*, 574.

[17] J. Li, J. Wu, S. Li, G. Liu, Y. Cui, Z. Dong, H. Liu, X. Sun, *ChemSusChem* **2021**, *14*, 2721.

[18] S. He, A. Wei, W. Li, X. Bai, L. Zhang, X. Li, R. He, L. Yang, Z. Liu, *Electrochim. Acta* **2019**, *318*, 362.
